# Anesthetic State-Dependent Bidirectional Control of States of Consciousness via Heterogeneous Medial Septum to Ventral Tegmental Area Circuits under Sevoflurane in Mice

**DOI:** 10.1523/JNEUROSCI.1383-25.2026

**Published:** 2026-03-06

**Authors:** Jia-Yi Wu, Chi Cui, Xin-Yi Dai, Wei Wang, Dai-Qiang Liu, Hong Wu, Shao-Jie Gao, Long-Qing Zhang, Lin Liu, Wen-Lu Song, Ya-Qun Zhou, Pei Zhang, Bo Tian, Shi-Ling Chen, Dan-Yang Chen, Ping Zhang, Wei Mei, Zhou-Ping Tang

**Affiliations:** ^1^Department of Anesthesiology and Pain Medicine, Hubei Key Laboratory of Geriatric Anesthesia and Perioperative Brain Health, and Wuhan Clinical Research Center for Geriatric Anesthesia, Tongji Hospital, Tongji Medical College, Huazhong University of Science and Technology, Wuhan 430030, China; ^2^School of Medicine, Wuhan University of Science and Technology, Wuhan 430081, China; ^3^ Departments of Physiology, Tongji Medical College, Huazhong University of Science and Technology, Wuhan 430030, China; ^4^ Neurobiology, School of Basic Medicine, Tongji Medical College, Huazhong University of Science and Technology, Wuhan 430030, China; ^5^Department of Neurology, Tongji Hospital, Tongji Medical College, Huazhong University of Science and Technology, Wuhan 430030, China; ^6^Department of Neurology, Tongji Hospital, Tongji Medical College and State Key Laboratory for Diagnosis and Treatment of Severe Zoonotic Infectious Diseases, Huazhong University of Science and Technology, Wuhan 430030, China

**Keywords:** consciousness, general anesthesia, medial septum, sevoflurane, ventral tegmental area

## Abstract

General anesthesia (GA) induces reversible unconsciousness for surgery, yet mechanisms underlying bidirectional transitions of states of consciousness during GA remain largely unknown. Here, we focused on states of consciousness rather than contents of consciousness, which reflects the capacity for responsiveness to stimuli. Electroencephalography/electromyography was applied in both male and female mice to investigate states of consciousness during sevoflurane GA. We identified the population activity of glutamatergic neurons in the medial septum (MS) to change synchronously with altered states of consciousness during sevoflurane GA. Activation of glutamatergic MS neurons (MS^Vglut2^) or their projections in the ventral tegmental area (VTA) facilitated behavioral emergence and cortical activation during sevoflurane GA, while their inhibition deepened cortical inhibition. Nevertheless, we further identified anesthetic state-dependent dual control of states of consciousness by monosynaptic innervations from MS^Vglut2^ neurons to heterogeneous downstream VTA neurons. Specifically, optogenetic activation of MS-innervated glutamatergic VTA neurons promoted cortical activation during both continuous steady-state GA (CSSGA) and burst suppression (BS). With current stimulation protocol, optogenetic activation of MS-innervated dopaminergic VTA neurons promoted cortical activation mainly under CSSGA. Optogenetic activation of MS-innervated GABAergic VTA neurons enhanced cortical inhibition mainly under BS. Our findings reveal an anesthetic state-dependent mechanism where MS^Vglut2^ neurons bidirectionally regulate states of consciousness through heterogeneous VTA neurons, providing insights to the complexity in the regulation of states of consciousness under GA.

## Significance Statement

While neuronal circuits modulating general anesthesia (GA) are increasingly mapped, bidirectional control of states of consciousness by the same neuronal ensemble and its anesthetic state dependence remain overlooked. We reveal heterogeneous MS (medial septum)–VTA (ventral tegmental area) circuits where glutamatergic MS neurons recruit distinct VTA subpopulations to bidirectionally regulate states of consciousness: downstream glutamatergic neurons promote cortical activation during both light and deep GA, and downstream dopaminergic neurons promote cortical activation mainly during light GA, while downstream GABAergic populations enhance cortical inhibition during deep GA. Our findings demonstrate subcortical complexity in the regulation of states of consciousness, offering novel targets for precise anesthetic control.

## Introduction

General anesthesia (GA), one of the most important medical advances of the millennium, is indispensable for modern surgical interventions for its ability to induce reversible unconsciousness (Editorial, 2000; Mashour, 2024). In the past decades, great progress has been made on neurobiological mechanisms underlying GA-induced changes in state/level of consciousness (rather than content of consciousness). Here, state/level of consciousness refers to the capacity for responsiveness to stimuli; this term, rather than “arousal”, was adopted to account for conditions such as sleepwalking, where individuals may respond to verbal requests despite nonaroused states (Idir et al., 2025). Numerous neurons and neural circuits have been identified to be either emergence-promoting or GA-induced unconsciousness-promoting (Brown et al., 2011; Hemmings et al., 2019; Bao et al., 2023). However, how these opposing mechanisms coordinate to regulate bidirectional states of consciousness transitions across different anesthetic depths remains poorly understood (Mashour, 2024). Inappropriate anesthetic depth may cause complications (Gareev et al., 2025; Kajiwara et al., 2025), highlighting the importance of elucidating the specific mechanisms underlying bidirectional states of consciousness transitions under GA.

We focus on the medial septum (MS), a basal forebrain structure involved in locomotion (Fuhrmann et al., 2015), exploration (Gangadharan et al., 2016), feeding (Sweeney et al., 2017), reward (Shen et al., 2022), aversion (Zhang et al., 2018), learning, and memory (Griguoli and Pimpinella, 2022; Kiraly et al., 2023). Notably, MS also regulates sleep–wakefulness, with glutamatergic MS neurons (MS^Vglut2^) proven to be wake-active and wake-promoting (Lu et al., 2020; An et al., 2021; Liang et al., 2023). Furthermore, electrolytic lesions of MS or pharmacological lesions of cholinergic MS neurons increase sensitivity to and delay emergence from isoflurane GA (Leung et al., 2013; Tai et al., 2014). These findings suggest that MS may also participate in the regulation of states of consciousness during sevoflurane GA, with MS^Vglut2^ neurons being the potential ensemble.

It is demonstrated that MS^Vglut2^ neurons project to the ventral tegmental area (VTA; An et al., 2021), a heterogeneous structure in the midbrain, containing glutamatergic, dopaminergic, and GABAergic neurons (Nair-Roberts et al., 2008; Pignatelli and Bonci, 2015; Morales and Margolis, 2017). Apart from its well-known effects on reward and aversion, VTA plays important roles in regulating sleep–wakefulness states (Eban-Rothschild et al., 2016; Chowdhury et al., 2019; Yu et al., 2019) and states of consciousness during isoflurane GA (Taylor et al., 2016; Yin et al., 2019; Zhang et al., 2024). Intriguingly, while all these three VTA populations are wake-active, they exhibit different functions: glutamatergic and dopaminergic VTA neurons are wake-promoting, whereas GABAergic VTA neurons are nonrapid eye movement (NREM) sleep-promoting (Eban-Rothschild et al., 2016; Chowdhury et al., 2019; Yu et al., 2019). Similarly, during isoflurane GA, glutamatergic and dopaminergic VTA neurons are demonstrated to be emergence-promoting (Taylor et al., 2016; Yin et al., 2019), while GABAergic VTA neurons are identified to facilitate unconsciousness (Zhang et al., 2024). However, given emerging evidence that sevoflurane and isoflurane may differ in the neural mechanisms for inducing unconsciousness (Yi et al., 2023), whether these VTA populations play similar roles in regulating states of consciousness during sevoflurane GA remains to be explored. Additionally, the specific VTA neurons innervated by MS^Vglut2^ neurons and their potential role in modulating states of consciousness under sevoflurane GA remain unknown.

In the current study, anterograde and retrograde tracing, in vivo fiber photometry, chemogenetic and optogenetic manipulations, and electroencephalography/electromyography (EEG/EMG) were employed to investigate the role of MS^Vglut2^ neurons and the MS^Vglut2^–VTA pathway in regulating states of consciousness under sevoflurane GA. Rabies-mediated retrograde tracing was applied to identify innervations from MS^Vglut2^ neurons to three types of VTA neurons (glutamatergic, dopaminergic, and GABAergic). Specific optogenetic stimulations of these three types of MS–VTA circuits were further carried out by anterograde trans-synaptic adeno-associated virus (AAV) vectors. We demonstrate that MS^Vglut2^ neurons exert anesthetic state-dependent bidirectional control on states of consciousness through heterogeneous downstream VTA populations during sevoflurane GA.

## Materials and Methods

### Animals

Adult (8–10 weeks) C57BL/6J mice, Vglut2-Cre mice, DAT-Cre mice, or Vagt-Cre mice were used. In this study, male and female mice were equally distributed across the groups. All mice were group-housed (4–5 mice in a cage) based on gender, with *ad libitum* access to food and water. The pathogen-free facility was maintained at a constant ambient temperature (22°C [1°C]) and humidity (50% [5%]) under a stable 12 h light/dark cycle (illumination intensity ≈ 300 lux, lights on at 08:00). All experimental procedures involving animals were approved by the Animal Experimentation Ethics Committee of Tongji Hospital, Huazhong University of Science and Technology (TJH-202312020). In this study, online randomization tools (https://www.random.org/lists/) were applied to assign animals to each group. The animals were tested in a sequential order, and the experimenters conducting all behavior tests were blinded to group allocations.

### Virus

AAV2/9-hSyn-FLEX-GCaMP8f, AAV2/9-CaMKIIα-hM3Dq-mCherry, AAV2/9-CaMKIIα-hM4Di-mCherry, AAV2/9-CaMKIIα-mCherry, AAV2/9-Ef1α-DIO-ChR2-mCherry, AAV2/9-Ef1α-DIO-eNpHR3.0-mCherry, AAV2/9-Ef1α-DIO-mCherry, AAV2/9-CaMKIIα-ChR2-mCherry, and AAV2/9-CaMKIIα-eNpHR3.0-mCherry were purchased from Obio Technology. AAV2/9-Ef1α-DIO-TVA-eGFP, AAV2/9-Ef1α-DIO-RVG, RV-EnvA-ΔG-DsRed, AAV2/1-CaMKIIα-Cre (1.42 × 10^13^ v.g./ml), AAV2/1-TH-Cre (1.81 × 10^13^ v.g./ml), and AAV2/1-GAD67-Cre (1.43 × 10^13^ v.g./ml) were obtained from BrainVTA. All viruses were aliquoted and stored at −80°C until use.

### Surgery

Mice were anesthetized with sodium pentobarbital (50 mg/kg, i.p.) prior to placement on the stereotactic frame (RWD Life Science). The body temperature of the mice was maintained at 36–37°C by a far-infrared heating pad (Kent Scientific) throughout the operation. An additional subcutaneous injection of bupivacaine 0.5% was administered for analgesia before a longitudinal incision was made to expose the skull. Then the mice were given a small craniotomy hole above the target region, and the virus was injected into the target region via a glass pipette using a stereotaxic injector (Stoelting) at a rate of 40 nl/min. After the injection, the glass pipette was left at the injection site for another 10 min. Stereotaxic coordinates and injection details were as follows: MS, AP +0.65 mm, ML +0.55 mm, and DV −3.95 mm, with an 8° angle toward the midline and a volume of ∼200 nl injected unilaterally, and VTA, AP −3.35 mm, ML ±1.15 mm, and DV −4.30 mm, with an 8° angle toward the midline and a volume of ∼100 nl/side injected bilaterally. For RV-mediated retrograde tracing of VTA neurons, a mixture of two helper AAVs was bilaterally injected into the VTA. Then RV-EnvA-ΔG-DsRed was injected into the same region 21 d later. Seven days later, the mice were killed.

Two weeks after the virus injection, the optic fiber (200 µm, 0.37 NA, Inper) was implanted 200 µm above the target region. For in vivo fiber photometry of the MS^Vglut2^–VTA projections, optogenetic stimulation of the MS^Vglut2^–VTA projections, or optogenetic stimulation of Glu^MS–VTA^ neurons, the optic fiber was unilaterally implanted into the VTA (AP −3.35 mm, ML +0.15 mm, DV −4.10 mm). For optogenetic inhibition of the MS^Vglut2^–VTA projections, optogenetic stimulation of DA^MS–VTA^ neurons, or optogenetic stimulation of GABA^MS–VTA^ neurons, the optic fiber was bilaterally implanted into the VTA (AP −3.35 mm, ML ±1.15 mm, DV −4.15 mm). The EEG/EMG electrode was embedded in the same surgery of optic fiber implantation. Specifically, two stainless steel screws of EEG electrodes were respectively fixed in the right prefrontal cortex (recording electrode, AP +1.75 mm, ML −0.4 mm) or the left cerebellum (reference electrode), and two EMG electrodes were inserted into the neck muscle.

### Fiber photometry recording and analysis

In vivo fiber photometry recording was conducted as previously described (Bao et al., 2021; Wu et al., 2025). The mice were placed into an acrylic glass chamber for prehabituation lasting 2 h a day for 3 consecutive days before the recording. On the recording day, the implanted optic fiber was connected to the fiber photometry system (Inper) through an optical fiber patch cord (Inper), and the mice were placed into the acrylic glass chamber with 100% oxygen at 1 L/min to habituate for another 30 min. During fiber photometry recording, EEG/EMG recording was conducted simultaneously. The recording started with baseline recording for 15 min, then sevoflurane (1.5 or 2.4% with oxygen 100%) was delivered (or only oxygen 100% as control) for 15 min. After turning off the sevoflurane, the recoding was continued for another 15 min. The concentration of sevoflurane was monitored in real time by an anesthetic agent analyzer (G60, Philips). In brief, the Ca^2+^ signals were recorded for a total of 45 min (15 min each for before, during, and after the delivery of sevoflurane), and the fluorescence changes of these three periods were calculated separately. A heating pad was placed under the chamber to maintain the body temperature of mice at 37°C throughout experiments.

Based on previous studies (Jiang-Xie et al., 2019; Yi et al., 2023; Wu et al., 2025), we established a multiparametric criterion for identifying the timepoint of loss of consciousness (LOC) and recovery of consciousness (ROC). Specifically, we firstly calculated the average delta power during the baseline (300 s before the administration of sevoflurane). The timepoint of LOC was then defined as the moment when the following three conditions were met: (1) the delta power during induction firstly exceeded the baseline average, (2) the theta/delta ratio firstly decreased to 0.3, and (3) EMG activity remained continuously minimal for 60 s. Similarly, the timepoint of ROC was determined based on (1) the average delta power of the 300 s before the discontinuation of sevoflurane, (2) theta/delta ratio firstly increasing to 0.3, and (3) increased EMG activity. Based on the timepoint of LOC and the timepoint of the onset of 2.4% sevoflurane (defined as 0 s), the induction phase (−180 to +300 s) was separated into three consecutive periods: baseline (b.s.), −180 to 0 s; pre-LOC, 0 s to LOC; and post-LOC, LOC to +300 s. Similarly, according to the timepoint of the shutoff of 2.4% sevoflurane (defined as 0 s) and the timepoint of ROC, the emergence phase (−180 to +300 s) can also be separated into three consecutive periods: b.s., −180 to 0 s; pre-ROC, 0 s to ROC; and post-ROC, ROC to +300 s. The fluorescence changes of the three consecutive periods during the induction or emergence phase were calculated separately. Likewise, since the mice did not exhibit typical LOC or ROC under 1.5% sevoflurane, the “induction” phase (−180 to +600 s, relative to the onset of sevoflurane) or the “emergence” phase (−180 to +600 s, relative to the shutoff of sevoflurane) under 1.5% sevoflurane was separated into following three consecutive periods: −180 to 0 s as baseline, 0 to +300 s, and +300 to +600 s.

Analysis of the raw Ca^2+^ signal was performed using Inper Data Process (V0.7.2, Inper). The fluorescence change (Δ*F*/*F*) was calculated as (*F* − *F*_baseline_) / *F*_baseline_, where *F*_baseline_ was the baseline fluorescence during the 15 min before sevoflurane delivery (in the whole-time analysis), during −180 to 0 s before sevoflurane delivery (in the analysis of the induction phase), or during −180 to 0 s before sevoflurane discontinuance (in the analysis of the emergence phase).

### Chemogenetic manipulations

As previously described (Bao et al., 2021; Wu et al., 2025), anesthesia behavioral experiments were conducted under chemogenetic manipulations. For habituation prior to experiments, the mice were placed into a cylindrical chamber with a 1 L/min flow of 100% oxygen for 2 h a day over 3 consecutive days. After habituation, clozapine-*N*-oxide (CNO; 3 mg/kg, i.p., BrainVTA) or an equivalent volume of saline was administered to mice 1 h before sevoflurane inhalation. During behavioral experiments, the concentration of sevoflurane was continuously monitored by the anesthetic agent analyzer, and the temperature of the chamber was maintained at 37°C by the heating pad.

To determine the time to the loss of the righting reflex (LORR; defined as the mouse remaining in the supine position for >30 s), 1 L/min flow of 100% oxygen with 2.4% sevoflurane was delivered. The chamber was rotated 180° every 15 s to check for LORR. The time to LORR was defined as the interval between the timepoint of the delivery of 2.4% sevoflurane to the timepoint of LORR. After LORR, 2.4% sevoflurane was continued for another 30 min before the sevoflurane was turned off. The time to the recovery of the righting reflex (RORR) was defined as the interval between the timepoint of the discontinuance of sevoflurane to the timepoint when the mouse turned itself to the prone position.

To determine the dose–response curve for LORR, the initial concentration of sevoflurane was set at 0.9%, and the concentration was increased in increments of 0.1% every 15 min to allow for equilibration of the anesthetic vapor in the chamber. The chamber was rotated 180° at the end of each 15 min interval to assess LORR. The number of mice that showed LORR at each concentration was recorded, and the experiments ended when all mice had shown LORR. To determine the dose–response curve for RORR, all mice were firstly exposed to 2.4% sevoflurane for 30 min and then placed to the supine position. The concentration of sevoflurane was decreased in increments of 0.1% every 15 min. The number of mice that exhibited RORR at each concentration was recorded, and the experiments stopped when all mice had shown RORR.

### Optogenetic manipulations

As previously described (Bao et al., 2021; Lin et al., 2024; Wu et al., 2025), for habituation prior to EEG/EMG recordings, mice were placed into the anesthetic chamber with a 1 L/min flow of 100% oxygen for 2 h a day over 3 consecutive days. The implanted optic fiber was connected to the laser stimulator (Newdoon) through the optical fiber patch cord, and the implanted EEG/EMG electrode was connected to the recording cables via a rotary connector. The temperature of the chamber was maintained at 37°C by the heating pad.

For optogenetic manipulations during continuous steady-state GA (CSSGA), after the recording of awake EEG/EMG for 5 min as baseline, mice were firstly exposed to 2.4% sevoflurane in 100% oxygen for 20 min and then placed to the supine position. The sevoflurane concentration was then decreased to 1.4%. If the mouse showed any signs of RORR, the concentration would be increased by 0.1% increments till the mouse remained on its supine position for 20 min consecutively at a constant concentration. In this study, the sevoflurane concentration ranged from 1.4 to 1.6% due to individual differences in mice. Pulses of blue laser with 10 ms width (473 nm, ∼5 mW, 10 Hz for MS^Vglut2^ neuronal stimulation, 10 or 20 Hz for MS–VTA axonal stimulation, and 20 Hz for MS-innervated VTA neuronal stimulation) or constant yellow laser (589 nm, ∼10 mW) were delivered for 2 min under CSSGA. Video recording was conducted during optogenetic manipulations to investigate arousal responses.

For optogenetic manipulations during burst suppression (BS), after a 5 min baseline EEG/EMG recording, the mouse was exposed to 2.4% sevoflurane in 100% oxygen. When the mouse had shown stable BS oscillations for at least 5 min, blue laser pulses (473 nm, ∼5 mW, 10 ms pulse) or constant yellow laser (589 nm, ∼10 mW) were delivered for 1 min.

The selection of the stimulation frequencies was based on previous similar studies targeting MS (An et al., 2021) or VTA (Eban-Rothschild et al., 2016; Yu et al., 2019). Besides, since the optogenetic stimulation intensity and frequency required for reliable activation between the somata and axonal terminals are different (Hay et al., 2011; Grossman et al., 2013; Rost et al., 2022), 10 and 20 Hz blue laser were used for MS–VTA axonal stimulation. In general, the natural firing patterns of these targeted neurons are as follows: MS^Vglut2^ neurons, ∼10 Hz (An et al., 2021); glutamatergic VTA neurons, ∼2 Hz (Miranda-Barrientos et al., 2021; Wang et al., 2021); dopaminergic VTA neurons, ∼2–4 Hz (Wang et al., 2021; Cui et al., 2025); and GABAergic VTA neurons, ∼10 Hz (Miranda-Barrientos et al., 2021; Wang et al., 2021).

### EEG/EMG recording and analysis

EEG/EMG recording and analysis were conducted as previously described (Liu et al., 2020; Lee et al., 2025; Wu et al., 2025). EEG signals were amplified by Microelectrode AC Amplifier Model 1700 (A-M Systems), analog bandpass-filtered at 0.1–500 Hz, and digitized at a sampling rate of 1,000 Hz via a data acquisition board (PCIe-6323, National Instruments) using the Spikehound software (Lott et al., 2009). The raw EEG data were analyzed using multitaper methods from the Chronux toolbox (version 2.1.2; http://chronux.org/) in MATLAB 2022b.

For EEG power spectral analysis, the raw EEG data were firstly bandpass-filtered at 0.5–50 Hz and then computed using a five-taper fast Fourier transform (FFT) for a window size of 4 s with 50% overlapping. For calculation of the power spectral density (PSD), the EEG data were divided into five frequency bands: delta (0.5–4 Hz), theta (4–10 Hz), alpha (10–15 Hz), beta (15–25 Hz), and gamma (25–50 Hz). For optogenetic manipulations under CSSGA, PSD were normalized by the average power at each frequency from 1 to 50 Hz to acquire a unitless ratio. For optogenetic manipulations during BS, the mean PSD of each single-frequency band was calculated over the entire frequency band (1–50 Hz). The choosing of normalized PSD or mean PSD was based on previous similar studies (Thordstein et al., 2004; Liu et al., 2020); specifically, the total EEG power remained relatively stable during CSSGA, while it decreased sharply during suppression periods of BS. The spectrogram, displayed using a decibel scale, showed the absolute power of EEG frequency bands.

For calculation of BS ratio (BSR), original EEG data were segregated into bursts or suppressions according to EEG voltage threshold and duration (Chemali et al., 2011). If the EEG signal exhibited amplitude of within ±15 μV and duration for at least 200 ms, it was then defined as a suppression period. If the interval of two adjacent suppression periods was <50 ms, these two periods were regarded as the same suppression period. The EEG epoch between suppression periods was regarded as a burst event. Suppressions or bursts were given a value of 1 or 0, respectively, to create a binary time series. This binary time series was then smoothed by a window function to calculate BSR over time.

### Arousal scoring

Arousal scoring was conducted as previously described (Bao et al., 2021; Wu et al., 2025). Via video recordings around the application of blue laser during CSSGA, the arousal responses of mice were scored by experimenters who were blinded to group allocations. Spontaneous movements of the head, tail, and limbs were scored as one of three levels: absent (0), mild (1), or moderate (2) in intensity. The righting behavior was scored as one of three levels: the mouse remained LORR with mild to moderate spontaneous movements (0), the mouse made an intense but unsuccessful attempt to righting itself (1), or the mouse successfully turned itself to the prone position (2). Walking after RORR was scored as follows: no further movements (0), crawled without the abdomen off the floor (1), or walked with the abdomen off the floor (2). The sum of all these items represented the arousal score of each mouse which had a maximum of 10.

### Whole-cell recordings

Whole-cell recordings of acute brain slices containing MS was conducted to verify the effectiveness of chemogenetic and optogenetic viruses, as previously described. After 3–4 weeks of viral expression, the mice were anesthetized and then decapitated. The brain was quickly removed and immersed in ice-cold, preoxygenated slice-cutting solution containing the following (in mM): 125 NaCl, 3.5 KCl, 25 NaHCO_3_, 1.25 NaH_2_PO_4_, 0.1 CaCl_2_, 3 MgCl_2_, and 10 glucose. Coronal sections (250 µm) containing MS were made with a vibratome (VT1000S, Leica). The slices were stored at 32°C for 1 h and then quickly transferred to ice-cold artificial cerebrospinal fluid, which consisted of the following (in mM): 125 NaCl, 25 NaHCO_3_, 1.25 NaH_2_PO_4_, 3.5 KCl, 2 CaCl_2_, 1 MgCl_2_, and 10 glucose, prefilled with 95% O_2_ and 5% CO_2_ (saturated with 95% O_2_ and 5% CO_2_). MS neurons expressing mCherry were clamped with borosilicate glass micropipettes (4–7 MΩ) under a microscope. The pipette intracellular solution contained the following (in mM): 140 K-gluconate, 13.4 Na-gluconate, 0.5 CaCl_2_, 1.0 MgCl_2_, 5 EGTA, 10 HEPES, 3 Mg^2+^-ATP, and 0.3 Na^+^-GTP, pH 7.4 (280–290 mOsm). Data were obtained using a MultiClamp 700B amplifier and pClamp 10 software (Molecular Devices), filtered at 2 kHz and sampled at a frequency of 2–10 kHz. The data were analyzed by the Clampfit 11.3 software (Molecular Devices).

For the validation of chemogenetic viruses, 5 µM CNO was applied to the bath for 2 min. For the verification of optogenetic viruses, blue laser (473 nm, ∼5 mW, 10 ms, 10 Hz) or constant yellow light (589 nm, ∼10 mW, 5 s) were given by the optical fiber placed above the cell.

### Histology

Immunofluorescence was performed as described previously (Wu et al., 2025). Briefly, mice were anesthetized and then perfused with phosphate-buffered saline (PBS) followed by ice-cold 4% paraformaldehyde (PFA). Brains were subsequently kept in 4% PFA at 4°C overnight and then successively placed in 20 and 30% sucrose dissolved in PBS at 4°C until they sank. Coronal sections of 20 µm thick of the brain were gained on a freezing microtome (CM1950, Leica), then mounted on poly-lysine-coated slides, and stored at −80°C till use. Brain slices were permeabilized with 0.3% Triton X-100 in PBS for 15 min and blocked with 10% goat serum for 1 h at room temperature. Then the primary antibody was incubated at 4°C overnight. Primary antibodies used in this study included rabbit monoclonal anti-Vglut2 antibody (1:200, catalog #ab216463, Abcam), rabbit monoclonal anti-c-Fos antibody (1:500, catalog #226003, Synaptic Systems), rabbit polyclonal anti-glutamate antibody (1:100, catalog #G6642, Sigma-Aldrich), rabbit polyclonal anti-TH antibody (1:200, catalog #A0028, ABclonal), and rabbit monoclonal anti-Vgat antibody (1:200, catalog #ab308062, Abcam). After rinsing five times in PBS for 6 min each, brain slices were incubated with secondary antibody for 2 h at room temperature in the dark. The secondary antibody used is FITC conjugated goat anti-rabbit IgG (H + L; 1:300, catalog #GB22303, ServiceBio). After rinsing five times in PBS for 8 min each, brain slices were incubated with DAPI (AR1177, Boster Biological Technology) for 5 min. The sections were coverslipped with fluorescent mounting medium and stored at 4°C before imaging. All images were acquired with an automated slide scanner system (VS120, Olympus).

For all mice underwent stereotactic surgery, those with incorrect virus expression ranges and (or) incorrect optic fiber implantations were excluded from data analysis.

For c-Fos staining in mice transfected with chemogenetic viruses in the MS, mice were given CNO (3 mg/kg, i.p.) or an equivalent volume of normal saline 90 min before anesthesia and perfusion.

### Statistical analysis

Data are presented as the mean ± SEM. All data underwent Shapiro–Wilk normality tests before analysis. Parametric or nonparametric tests were used according to the results of the normality tests. For parametric tests, paired or unpaired two-tailed Student's *t* tests, one-way or two-way repeated–measure ANOVA followed by Tukey's post hoc comparison tests were used, where appropriate. For nonparametric tests, Mann–Whitney rank-sum tests, Wilcoxon signed-rank tests, and Friedman tests followed by Dunn's post hoc comparison tests were used, where appropriate. The dose–response curve was generated by nonlinear regression with a variable slope (multiple parameters) to estimate the MAC of LORR or RORR and the corresponding 95% CI. Difference of MAC_LORR_ or MAC_RORR_ was tested with hypothesis testing using the overlap of range-preserving confidence intervals (Noguchi and Marmolejo-Ramos, 2016). A Bayesian Monte Carlo method was performed to evaluate the efficacy of optogenetic stimulation for restoring righting during CSSGA, as previously described (Taylor et al., 2016; Bao et al., 2021). The posterior probability of the difference in the probability to right between the ChR2-on group and mCherry-on group was derived from the beta distribution. It was considered statistically significant if the posterior probability was greater than 0.95 and the Bayesian CIs did not include 0. GraphPad Prism 9.0 (GraphPad Software) and MATLAB 2022b were used for statistical analyses. In all cases, *p* < 0.05 was considered statistically significant. Statistical reports of all data were provided in Dataset S1.

## Results

### MS^Vglut2^ neurons were sevoflurane GA-inhibited and emergence-promoting

**Figure 1.**
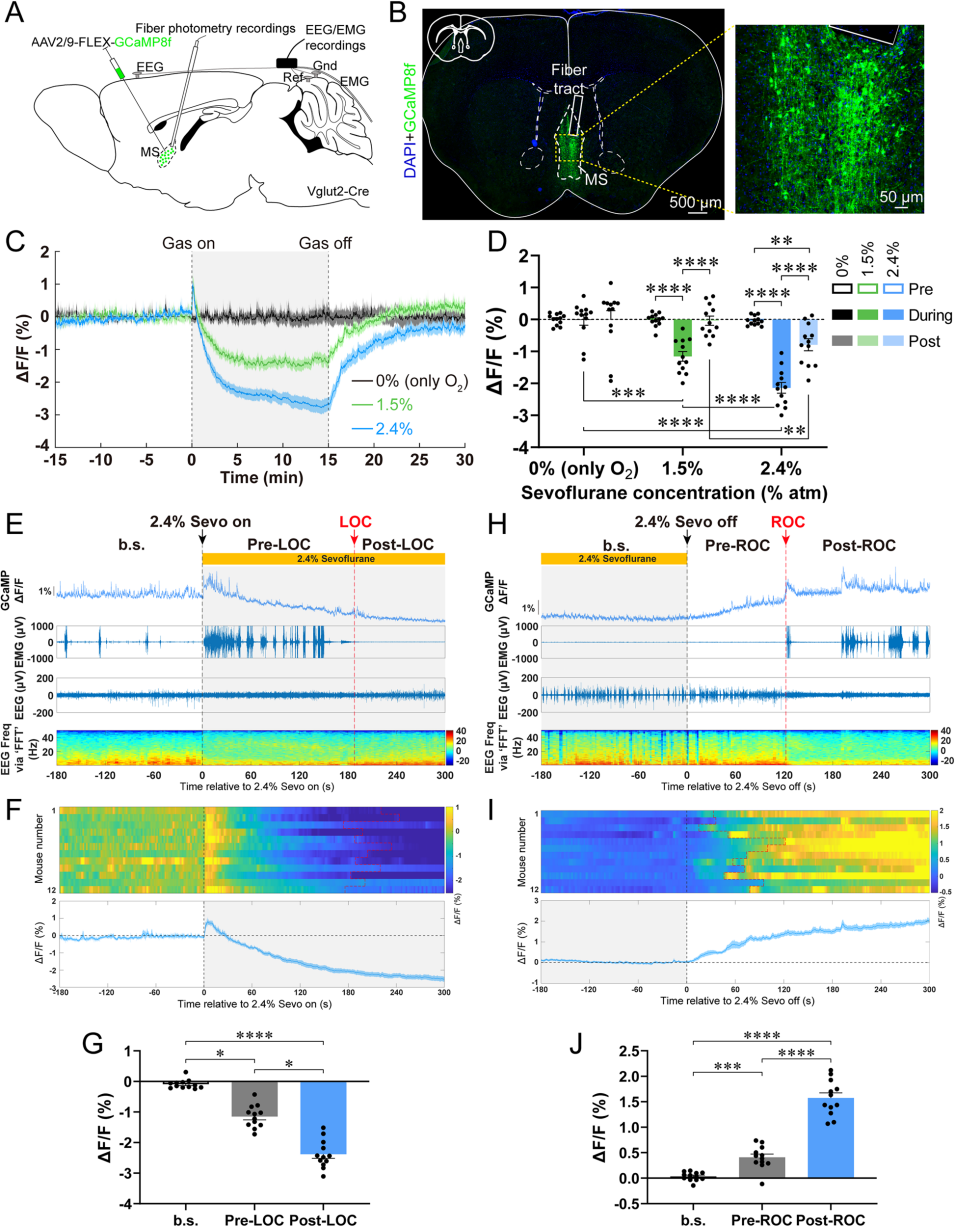
The population activity of MS^Vglut2^ neurons varies in synchrony with changes in states of consciousness under sevoflurane GA. ***A***, The configuration of in vivo fiber photometry recordings and EEG/EMG recordings. ***B***, Left, A representative image of GCaMP8f/DAPI immunofluorescence and the optic fiber tract. Right, Magnified images of the region of the yellow box. Scale bar: left, 500 μm; right, 50 μm. ***C***, Time courses of Ca^2+^ signals under different doses of sevoflurane. ***D***, Quantification of Ca^2+^ signal changes before, during, and after sevoflurane inhalation (*n* = 12 mice, mean ± SEM; statistical analysis was performed on data from the entire 45 min period, encompassing 15 min each for before, during, and after the delivery of sevoflurane). ***E***, A representative time courses of Ca^2+^ signals aligned with EEG/EMG state transitions of one mouse (from top to bottom: Ca^2+^ signal, EMG trace, EEG trace, EEG power spectrogram via FFT). Notice a gradual change of the consciousness level; also see Figures S1–S3. ***F***, Heatmaps (top) and mean GCaMP8f time courses ± SEM (shaded area; bottom) of all 12 mice during the time window relative to the onset of sevoflurane. ***G***, Quantification of Ca^2+^ signal changes in three consecutive time sections around LOC (*n* = 12 mice). ***H–J***, Similar to ***E*–*G*** but for the time window relative to the shutoff of 2.4% sevoflurane (*n* = 12 mice). Statistical comparisons were conducted using two-way repeated–measure ANOVA followed by Tukey's post hoc comparison tests (***D***), Friedman tests followed by Dunn's post hoc comparison tests (***G***), or one-way repeated–measure ANOVA followed by Tukey's post hoc comparison tests (***J***). *p < 0.05; **p < 0.01; ***p < 0.001; ****p < 0.0001. All error bars indicate ±SEM.

Firstly, we applied in vivo fiber photometry of MS^Vglut2^ neurons under different doses of sevoflurane (Fig. 1*A*,*B*; Fig. S1*A*). There was no significant change in the Ca^2+^ signal intensity in the 0% group (pure oxygen, as control; Fig. 1*C*,*D*). In contrast, the Ca^2+^ signal intensity significantly decreased under 1.5 and 2.4% sevoflurane (Fig. 1*C*,*D*). In addition, compared with the 1.5% group, the Ca^2+^ signal intensity significantly reduced when exposed to 2.4% sevoflurane (Fig. 1*C*,*D*). We further analyzed the Ca^2+^ signal intensity of MS^Vglut2^ neurons during the induction and emergence phases of sevoflurane GA, respectively. During the induction, the Ca^2+^ signal intensity gradually decreased upon administration of 2.4% sevoflurane, and it reached an even lower level after the LOC (Fig. 1*E*–*G*; Figs. S1*B*–*D*, S2*A*,*B*). As to the emergence phase, the Ca^2+^ signal intensity started to climb back steadily after the end of 2.4% sevoflurane delivery, and it dramatically rose to a higher level once the ROC showed (Fig. 1*H–J*; Figs. S1*E*,*F*, S2*C*,*D*). Similar results were found under 1.5% sevoflurane; the Ca^2+^ signal intensity changes synchronously with the gradual change of the level of consciousness (Fig. S3). These results indicate that the population activity of MS^Vglut2^ neurons is dose-dependently inhibited by sevoflurane and is in synchrony with changes of states of consciousness under sevoflurane GA.

**Figure 2.**
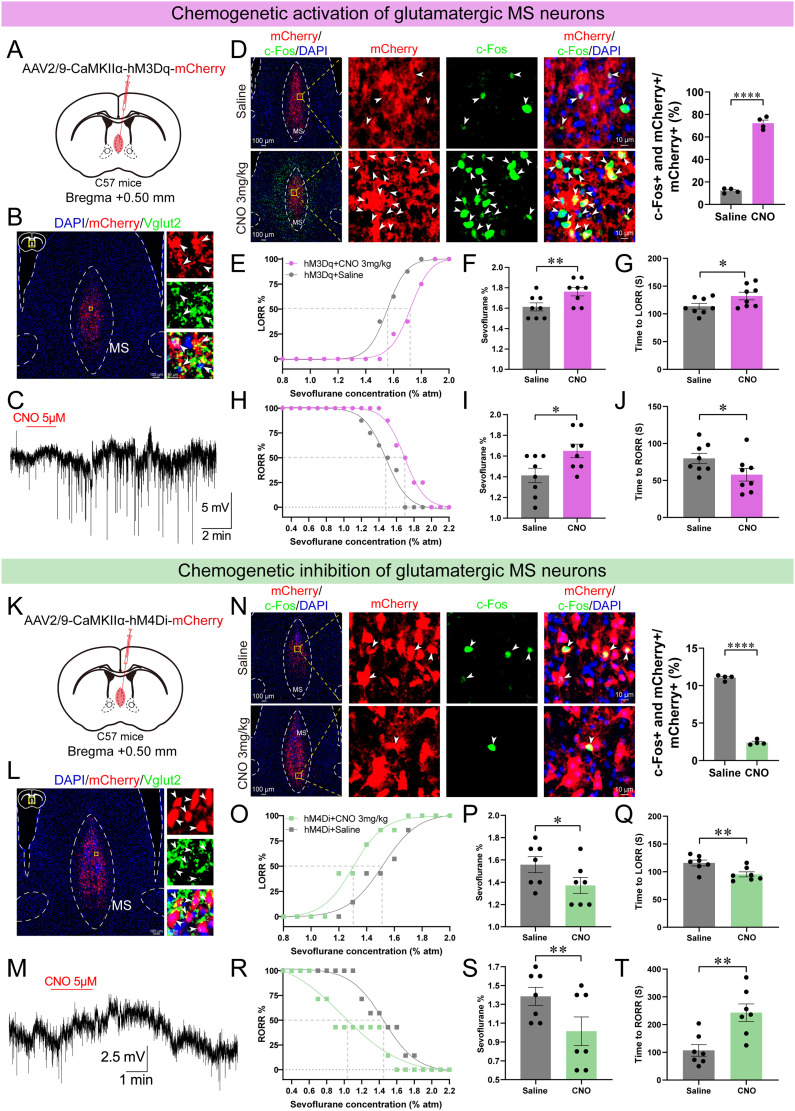
Chemogenetic activation or inhibition of MS^Vglut2^ neurons decreases or enhances the sensitivity to sevoflurane GA in both induction and emergence phases, respectively. ***A***, ***B***, Schematic of hM3Dq-mCherry injection (***A***) and expression (***B***) in MS^Vglut2^ neurons (arrowheads indicate colabeled neurons; scale bar, left, 100 μm; right, 10 μm). ***C***, Whole-cell recordings showing that 2 min bath application of CNO (5 μM) increased the activity of hM3Dq-mCherry–positive MS^Vglut2^ neurons in vitro. ***D***, Left, Representative images of the overlap expression of hM3Dq-mCherry, c-Fos, and DAPI in the MS after CNO (3 mg/kg, i.p.) or saline treatment (solid arrowheads indicate hM3Dq-mCherry and c-Fos colabeled neurons; scale bar, left, 100 μm; right, 10 μm). Right, Percentage of hM3Dq-mCherry and c-Fos colabeled cells in hM3Dq-mCherry–positive cells (*n* = 4 mice). ***E–G***, Dose–response curves of the percentage of (***E***), the sevoflurane concentration of (***F***), and the time to (***G***) LORR of hM3Dq mice after CNO (3 mg/kg, i.p.) or saline treatment (*n* = 8 mice). ***H–J***, Similar to ***E*–*G*** but displaying the dose–response curves of (***H***), the sevoflurane concentration of (***I***), and the time to (***J***) RORR of hM3Dq mice (*n* = 8 mice). ***K–M***, Schematic of injection (***K***), virus expression (***L***), and whole-cell recordings (***M***) of hM4Di AAV vector (***L***, arrowheads indicate colabeled neurons; scale bar, left, 100 μm; right, 10 μm. ***M***, 2 min bath of 5 μM CNO). ***N***, Left, Representative images of the overlap expression of hM4Di-mCherry, c-Fos, and DAPI in the MS after CNO (3 mg/kg, i.p.) or saline treatment (solid arrowheads indicate hM4Di-mCherry and c-Fos colabeled neurons; scale bar, left, 100 μm; right, 10 μm). Right, Percentage of hM4Di-mCherry and c-Fos colabeled cells in hM4Di-mCherry–positive cells (*n* = 4 mice). ***O–T***, Similar to ***E*–*J*** but displaying the data of behavioral tests in hM4Di mice (*n* = 7 mice). Statistical comparisons were conducted using unpaired (***D***, ***N***) or paired (***F***, ***G***, ***I***, ***J***, ***P***, ***Q***, ***S***, ***T***) two-tailed Student's *t* test. *p < 0.05; **p < 0.01; ****p < 0.0001. All error bars represent ±SEM.

Chemogenetic bidirectional manipulations of MS^Vglut2^ neurons were then conducted to investigate their role during the induction and emergence phases of sevoflurane GA. The effectiveness of the hM3Dq (Gq) expression was confirmed by whole-cell recordings from acute brain slices and c-Fos staining (Fig. 2*A*–*D*; Fig. S4*A*). Chemogenetic activation of MS^Vglut2^ neurons increased the minimum alveolar concentration (MAC) for the LORR (MAC_LORR_; Fig. 2*E*,*F*), delayed the time to LORR (Fig. 2*G*), increased the MAC for the RORR (MAC_RORR_; Fig. 2*H*,*I*), and shortened the time to RORR (Fig. 2*J*). We further employed chemogenetic inhibition of MS^Vglut2^ neurons, the hM4Di (Gi) expression was also confirmed by whole-cell recordings and c-Fos staining (Fig. 2*K*–*N*; Fig. S4*B*). Chemogenetic inhibition of MS^Vglut2^ neurons decreased the MAC_LORR_ (Fig. 2*O*,*P*), shortened the time to LORR (Fig. 2*Q*), decreased the MAC_RORR_ (Fig. 2*R*,*S*), and delayed the time to RORR (Fig. 2*T*). Furthermore, in control mice expressing only mCherry but not Gq or Gi, no effect on the induction or emergence process of sevoflurane GA was observed (Fig. S4*C*–*F*). Together, these results indicate that chemogenetic activation of MS^Vglut2^ neurons decreases the sensitivity to, antagonizes the induction of, and facilitates the emergence from sevoflurane GA; on the contrary, inhibition of MS^Vglut2^ neurons increases the sensitivity to, facilitates the induction of, and antagonizes the emergence from sevoflurane GA.

**Figure 3.**
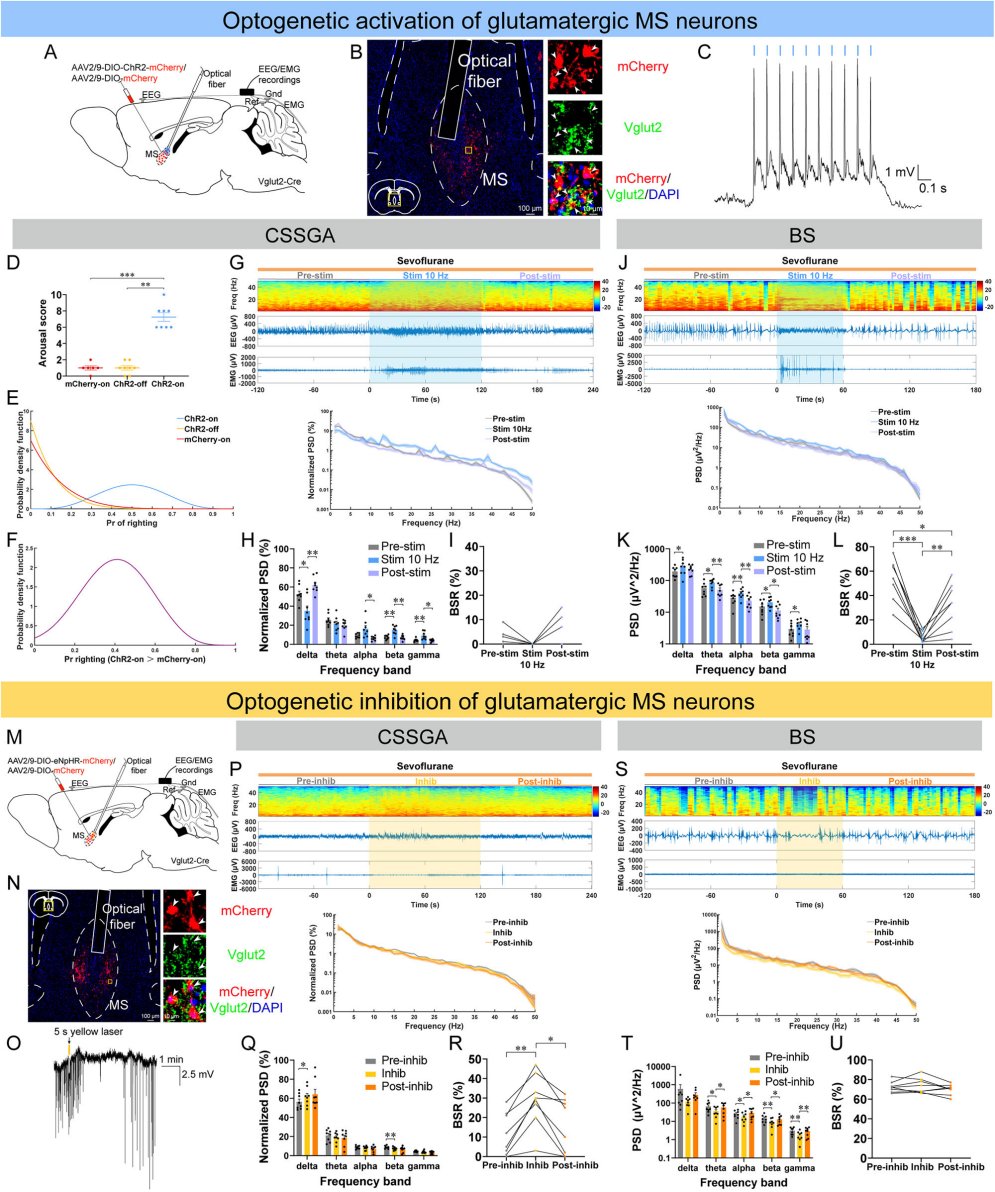
Optogenetic stimulation or inhibition of MS^Vglut2^ neurons decreases or deepens the depth of consciousness during CSSGA and BS with sevoflurane, respectively. ***A***, Configuration of optogenetic stimulation of MS^Vglut2^ neurons. ***B***, Left, A representative image of ChR2-mCherry expression and fiber tract. Right, An enlarged view of the region of the yellow box showing the overlap expression of ChR2-mCherry and Vglut2. Arrowheads indicate colabeled neurons. Scale bar: left, 100 μm; right, 10 μm. **C**, Whole-cell recordings showing that blue laser (473 nm, ∼5 mW, 10 ms, 10 Hz) elicited the firing of ChR2-mCherry–positive MS^Vglut2^ neurons in vitro. ***D–F***, Quantification of the arousal score of the three groups (***D***), posterior densities for the probabilities of righting for the three groups (***E***), and the difference in the probability of righting between the ChR2-on group and mCherry-on group (***F***) during optogenetic stimulation of MS^Vglut2^ neurons under sevoflurane CSSGA (*n* = 8 ChR2 mice; *n* = 6 mCherry mice). ***G***, Top, A representative example of EEG power spectrograms (top), EEG traces (middle), and EMG traces (bottom) of one ChR2 mouse around optogenetic stimulation of MS^Vglut2^ neurons during sevoflurane CSSGA. Bottom, Normalized PSD of EEG before (gray), during (blue), and after (lavender) optogenetic stimulation (*n* = 8 mice; the shading indicates SEM). ***H***, ***I***, Quantitative analysis of normalized PSD (***H***) and BSR (***I***) before, during, and after optogenetic stimulation in ChR2 mice during sevoflurane CSSGA (*n* = 8 mice). ***J–L***, Similar to ***G*–*I*** but displaying changes of EEG/EMG of ChR2 mice during BS (*n* = 8 mice). ***M–O***, Similar to ***A*–*C*** but showing the configuration (***M***), virus expression (***N***), and whole-cell recordings (***O***) of the eNpHR AAV vector (***N***, arrowheads indicate colabeled neurons; scale bar: left, 100 μm; right, 10 μm; ***O***, constant yellow laser, 589 nm, ∼10 mW, 5 s). ***P–R***, A representative example of EEG/EMG of one eNpHR mouse (***P***) and quantitative analysis of normalized PSD (***Q***) and BSR (***R***) before (gray), during (yellow), and after (orange) optogenetic inhibition during sevoflurane CSSGA (*n* = 8 mice; the shading indicates SEM). ***S–U***, Similar to ***P*–*R*** but displaying changes of EEG/EMG of eNpHR mice during BS (*n* = 8 mice). Statistical comparisons were conducted using unpaired two-tailed Student's *t* test (***D***, mCherry-on vs ChR2-off), Wilcoxon signed-rank test (***D***, ChR2-on vs ChR2-off), Mann–Whitney rank-sum test (***D***, mCherry-on vs ChR2-on), Friedman tests followed by Dunn's post hoc comparison tests (***I***), one-way (***L***, ***R***, ***U***) or two-way (***H***, ***K***, ***Q***, ***T***) repeated–measure ANOVA followed by Tukey's post hoc test. *p < 0.05; **p < 0.01; ***p < 0.001. All error bars indicate ±SEM.

We further applied optogenetic bidirectional manipulations of MS^Vglut2^ neurons coupled with EEG/EMG recordings across distinct states of sevoflurane GA. In this study, two anesthetic depths were systematically investigated: CSSGA and BS. CSSGA represented a light anesthetic depth in which mice could just maintain LORR (1.4–1.6% sevoflurane concentration with minor interindividual variability). By comparison, BS was a deep anesthetic depth characterized by stable BS oscillations on the EEG (2.4% sevoflurane concentration). The effectiveness of the ChR2 expression was confirmed by whole-cell recordings (Fig. 3*A*–*C*). During CSSGA, optogenetic stimulation (473 nm, 10 Hz, ∼5 mW, 10 ms pulse, 2 min) of MS^Vglut2^ neurons significantly induced behavioral arousal in the ChR2 group, including head, legs, and tail movements (8/8), righting (4/8), and walking (1/8; Fig. 3*D*; Movie 1). In contrast, in control mice, behavioral arousal could rarely be observed during optogenetic stimulation (Fig. 3*D*; Movie 2). The Bayesian 95% CI for the difference in the probability of righting between the ChR2-on group and mCherry-on group was 0.044–0.725 (Fig. 3*E*,*F*). The posterior probability of the difference in righting between the two groups >0 was statistically significant, with a value of 0.9853 (Fig. 3*E*,*F*).

**Movie 1.** Optogenetic stimulation of MS^Vglut2^ neurons induced behavioral arousal and cortical activation during sevoflurane CSSGA in a ChR2 mouse. [View online]

**Movie 2.** The same blue laser applied to MS^Vglut2^ neurons had little effect on the behavior or cortical state of a mCherry-control mice during sevoflurane CSSGA. [View online]

Moreover, during CSSGA, 10 Hz blue laser could induce a rapid transition of EEG states (from an anesthetic state of high-amplitude, low-frequency activity to an awake-like cortical state of low-amplitude, high-frequency activity) with enhanced EMG activity in ChR2 mice (Fig. 3*G*; Movie 1), but not in control mice (Fig. S4*G*; Movie 2). Analyses of PSD and BSR of EEG data were next conducted. In general, during CSSGA, optogenetic stimulation of MS^Vglut2^ neurons decreased delta power but increased power of alpha, beta, and gamma in ChR2 mice (Fig. 3*G*,*H*). The decrease of BSR induced by optogenetic stimulation during CSSGA was not significant (Fig. 3*I*). Similarly, during BS, 10 Hz blue laser (∼5 mW, 1 min) also induced a rapid transition of EEG states (from BS oscillations to an awake-like cortical state of low-amplitude, high-frequency activity) with profound EMG activity in ChR2 mice (Fig. 3*J*; Movie 3). Besides, during BS, optogenetic stimulation increased the power of wide-range bands from delta to gamma and significantly decreased BSR (Fig. 3*J*–*L*). The same 10 Hz blue laser had no significant effect on PSD or BSR in control mice during either CSSGA or BS (Fig. S4*G*–*I*,*M*–*O*).

**Movie 3.** Optogenetic stimulation of MS^Vglut2^ neurons induced behavioral arousal and cortical activation during sevoflurane BS in a ChR2 mouse. [View online]

On the contrary, during CSSGA, optogenetic inhibition (589 nm, ∼10 mW, 2 min) of MS^Vglut2^ neurons increased delta power, decreased beta power, and increased BSR (Fig. 3*M*–*Q*). During BS, even at the baseline with such high BSR, application of constant yellow laser (∼10 mW, 1 min) managed to deepen the cortical states (induced isoelectric activity for about half a minute), together with decreased power of wide-range bands from theta to gamma, although no significant difference in BSR were found (Fig. 3*S*–*U*). In control mice, no significant difference in PSD or BSR was found when applying the same yellow laser during CSSGA or BS (Fig. S4*J*–*L*,*P*–*R*). In summary, these results of optogenetic bidirectional manipulations verify that MS^Vglut2^ neurons are important for the regulation of states of consciousness during different depth of sevoflurane GA.

### MS^Vglut2^ neurons promoted emergence from sevoflurane GA partly through their projections to the VTA

To explore the downstream functional circuit of MS^Vglut2^ neurons, we conducted anterograde tracing of MS^Vglut2^ neurons (Fig. S5*A*). Similar to a previous study (An et al., 2021), the axons of MS^Vglut2^ neurons were found in many brain regions involved in sleep–wakefulness control or states of consciousness regulation under GA, such as the lateral habenular nucleus (Gelegen et al., 2018; Zhao et al., 2021), the lateral hypothalamus (LH; Kelz et al., 2008; Herrera et al., 2016; Li et al., 2019; Zhao et al., 2021; D. Wang et al., 2024), dorsomedial hypothalamic nucleus (Zhong et al., 2017; Wang et al., 2023), supramammillary nucleus (Wu et al., 2025), VTA (Taylor et al., 2016; Yin et al., 2019; Zhang et al., 2024), periaqueductal gray (D. Wang et al., 2024), and Edinger–Westphal nucleus (Yi et al., 2023; Fig. S5*B*).

Given that the VTA is well recognized in sleep–wakefulness control (Eban-Rothschild et al., 2016; Chowdhury et al., 2019; Yu et al., 2019) and states of consciousness regulation under GA (Taylor et al., 2016; Yin et al., 2019; Zhang et al., 2024), we next employed in vivo fiber photometry of MS^Vglut2^ neuronal projections in the VTA (referred to as MS^Vglut2^–VTA projections) under 2.4% sevoflurane (Fig. S5*C*,*D*). During the induction, the Ca^2+^ signal intensity of MS^Vglut2^–VTA projections significantly decreased (Fig. S5*E*,*F*). However, for the emergence phase, although the Ca^2+^ signal intensity of MS^Vglut2^–VTA projections tended to increase, no significant difference was found, with one mouse showing decreased Ca^2+^ signal intensity even after the end of 2.4% sevoflurane (Fig. S5*G*,*H*).

**Figure 4.**
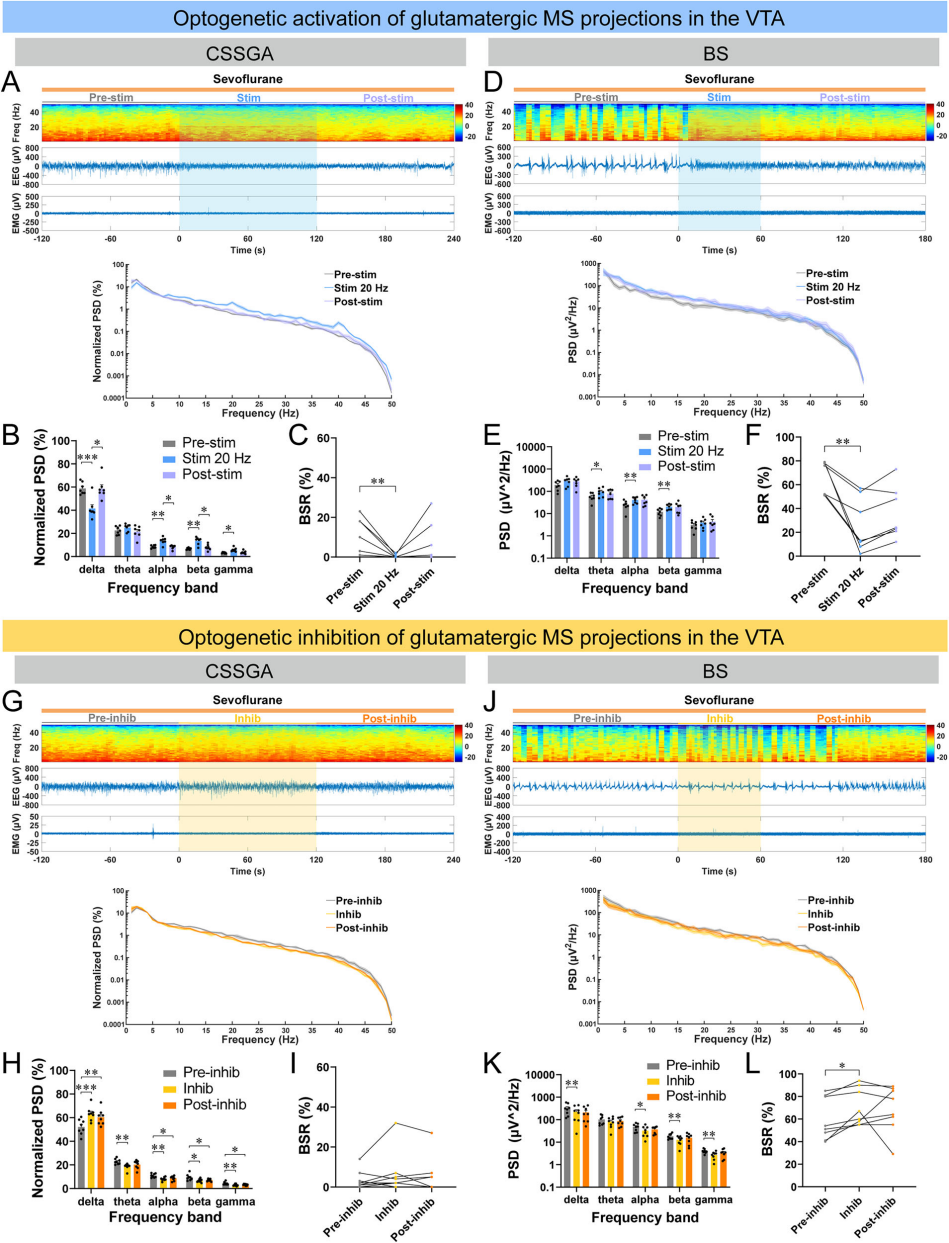
Optogenetic stimulation or inhibition of the MS^Vglut2^–VTA projections decreases or deepens the depth of consciousness during CSSGA and BS with sevoflurane, respectively. ***A*–*C***, A representative example of EEG/EMG of one mouse (***A***), quantitative analysis of normalized PSD (***B***), and BSR (***C***) before (gray), during (blue), and after (lavender) optogenetic stimulation of the MS^Vglut2^–VTA projections during sevoflurane CSSGA (*n* = 7 mice; the shading indicates SEM). ***D*–*F***, Similar to ***A–C*** but for optogenetic stimulation during BS (*n* = 7 mice). ***G*–*L***, Similar to ***A*–*F*** but displaying changes of EEG/EMG of eNpHR mice around optogenetic inhibition of the MS^Vglut2^–VTA projections during CSSGA (***G–I***) or BS (***J–L***) with sevoflurane (*n* = 8 mice). Statistical comparisons were conducted using Wilcoxon signed-rank test (***B***, ChR2-on vs ChR2-off), Mann–Whitney rank-sum test (***B***, mCherry-on vs ChR2-on and mCherry-on vs ChR2-off), Friedman tests followed by Dunn's post hoc comparison tests (***G***, ***J***, ***M***), one-way (***P***) or two-way (***F***, ***I***, ***L***, ***O***) repeated–measure ANOVA followed by Tukey's post hoc test. *p < 0.05; **p < 0.01; ***p < 0.001. All error bars represent ±SEM.

To further validate the potential role of MS^Vglut2^–VTA projections, optogenetic bidirectional manipulations of MS^Vglut2^–VTA projections were further conducted (Fig. S5*I*). Optogenetic stimulation of MS^Vglut2^–VTA projections by 20 Hz blue laser (∼5 mW, 10 ms pulse, 2 min) increased the arousal score in ChR2 mice (Fig. S5*J*; Movie 4), but not in control mice (Fig. S5*J*; Movie 5). The Bayesian 95% CI for the difference in the probability of righting between the ChR2-on group and mCherry-on group was −0.215 to 0.452; the posterior probability of the difference in righting between the two groups >0 was 0.7667 (Fig. S5*K*,*L*). Additionally, during CSSGA, optogenetic stimulation of MS^Vglut2^–VTA projections induced cortical activation, characterized by decreased delta power; increased power of alpha, beta, and gamma; and decreased BSR (Fig. 4*A–C*; Movie 4). Similarly, during BS, optogenetic stimulation of MS^Vglut2^–VTA projections also induced cortical activation (Fig. 4*D*–*F*). We also tested the efficacy of 10 Hz blue laser for the activation of the MS^Vglut2^–VTA projections, it turned out that 10 Hz stimulation could induce weaker effects than 20 Hz stimulation (Fig. S6*A*–*E*). On the contrary, optogenetic inhibition of MS^Vglut2^–VTA projections deepened cortical inhibition during both CSSGA and BS (Fig. 4*G–L*). In control mice, there was no significant difference in PSD or BSR when applied with the same blue or yellow laser under CSSGA or BS (Fig. S6*F*–*Q*). Together, these results suggest that, under different anesthetic states, the emergence-promoting effect of MS^Vglut2^ neurons was partly achieved through their projections to the VTA projections.

**Movie 4.** Optogenetic stimulation of MS^Vglut2^–VTA projections induced behavioral arousal and cortical activation during sevoflurane CSSGA in a ChR2 mouse. [View online]

**Movie 5.** The same blue laser applied to MS^Vglut2^–VTA projections had little effect on the behavior or cortical state of a mCherry-control mice during sevoflurane CSSGA. [View online]

### MS^Vglut2^ neurons monosynaptically innervate glutamatergic, dopaminergic, and GABAergic neurons in the VTA

**Figure 5.**
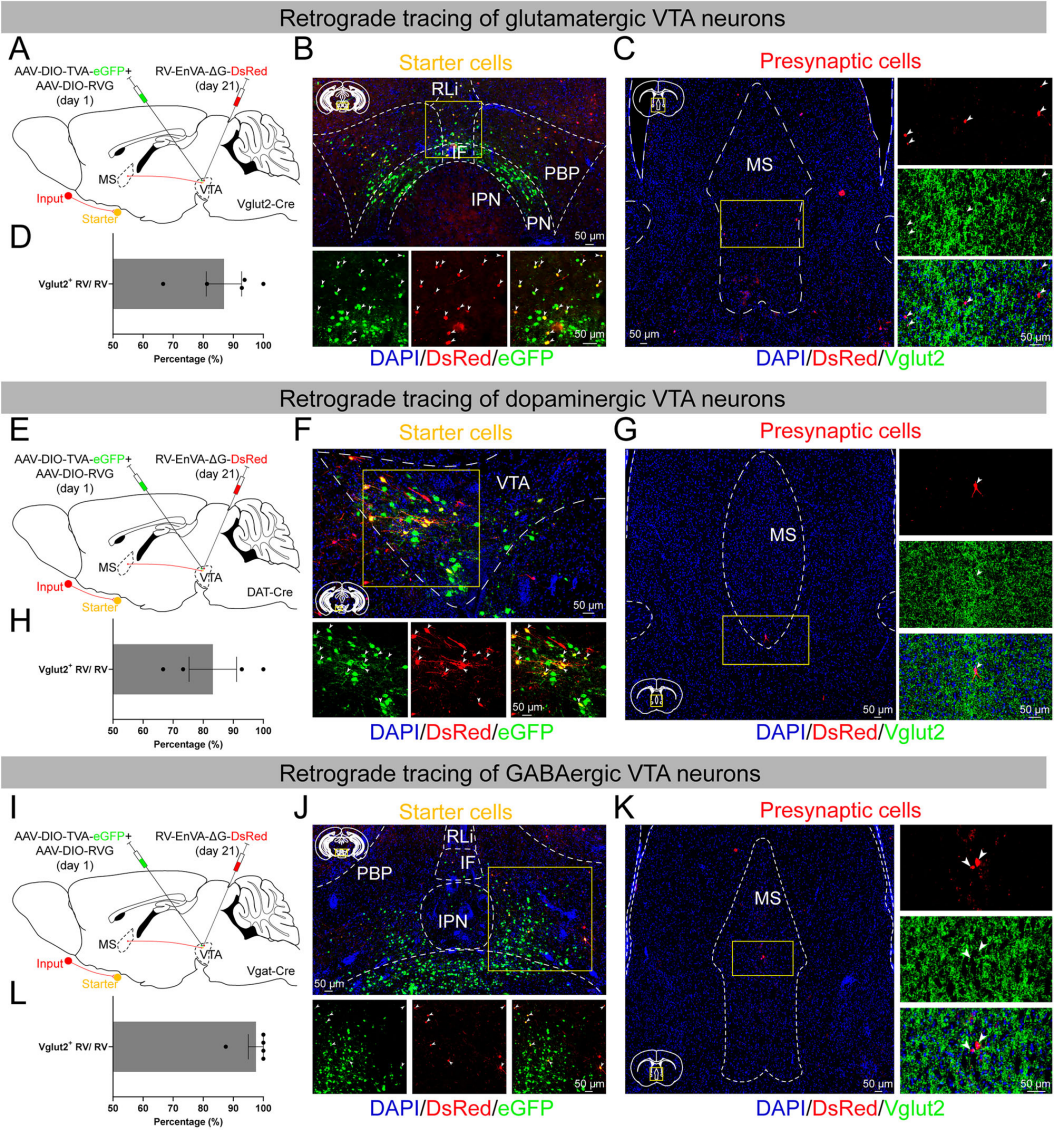
MS^Vglut2^ neurons form monosynaptic innervations to glutamatergic, dopaminergic, and GABAergic neurons in the VTA. ***A***, Schematic of retrograde monosynaptic tracing of VTA^Vglut2^ neurons. ***B***, Top, A representative image of the expression of helper virus (TVA-eGFP), RV (DsRed), and DAPI (blue) in the VTA. Bottom, An enlarged view of the region of the yellow box (arrowheads indicate the double-infected VTA^Vglut2^ neurons). Scale bar, 50 μm. ***C***, Left, A representative image of the expression of RV (DsRed) and DAPI (blue) in the MS. Right, An enlarged view of the region of the yellow box showing the neurons in the MS infected by RV (DsRed) were colabeled by Vglut2 (green). Scale bar, 50 μm. ***D***, The percentage of the RV and Vglut2 colabeled neurons in the RV-infected neurons in the MS (*n* = 5 mice). ***E–H***, Similar to ***A*–*D*** but displaying the results of retrograde monosynaptic tracing of dopaminergic VTA neurons (*n* = 4 mice). ***I*–*L***, Similar to ***A*–*D*** but displaying the results of retrograde monosynaptic tracing of GABAergic VTA neurons (*n* = 5 mice). All error bars indicate ±SEM.

Our results above showed that activation of the MS^Vglut2^–VTA projections could induce cortical activation during CSSGA and BS under sevoflurane. It's previously reported that glutamatergic and dopaminergic VTA neurons are both emergence-promoting (Taylor et al., 2016; Zhang et al., 2024), while GABAergic VTA neurons are GA-induced unconsciousness-promoting (Yin et al., 2019). To explore which neuronal population(s) in VTA was the downstream target(s) of GA-antagonizing MS^Vglut2^ neurons, we employed retrograde tracing of these three types of neurons in VTA by delivering the rabies virus (RV)-mediated retrograde trans-synaptic tracing system into the VTA of Vglut2-Cre, DAT-Cre, or Vgat-Cre mice. A mixture of two Cre-dependent helper AAV vectors (AAV2/9-DIO-TVA-eGFP and AAV2/9-DIO-RVG) was firstly injected into the VTA. After 3 weeks, RV-EnvA-ΔG-DsRed was injected into the same site of the VTA. Seven days after RV injection, the mice were killed for immunofluorescence staining (Fig. 5*A*,*E*,*I*).

In Vglut2-Cre mice, the TVA-eGFP and RV-DsRed double–infected glutamatergic starter cells were observed in VTA (Fig. 5*B*), with RV-infected presynaptic cells found in the MS (Fig. 5*C*). RV-infected presynaptic cells in the MS were almost colabeled by Vglut2, at the proportion of 86.90 ± 5.90%, indicating that glutamatergic VTA neurons receive monosynaptic inputs from MS^Vglut2^ neurons (Fig. 5*D*). In DAT-Cre mice, the TVA-eGFP and RV-DsRed double–infected dopaminergic starter cells were observed in VTA (Fig. 5*F*). RV-infected presynaptic cells in the MS were colabeled by Vglut2, at the proportion of 83.21 ± 7.89%, indicating that dopaminergic VTA neurons receive monosynaptic inputs from MS^Vglut2^ neurons (Fig. 5*G*,*H*). In Vgat-Cre mice, the TVA-eGFP and RV-DsRed double–infected GABAergic starter cells were observed in VTA (Fig. 5*J*), with RV-infected presynaptic MS cells colabeled by Vglut2 at the proportion of 97.50 ± 2.50%, suggesting that GABAergic VTA neurons also receive monosynaptic inputs from MS^Vglut2^ neurons (Fig. 5*K*,*L*). Together, all these three neuronal populations in VTA, glutamatergic, dopaminergic, and GABAergic neurons receive monosynaptic innervations from MS^Vglut2^ neurons.

To investigate the functions of these three MS-innervated VTA populations, we further conducted in vivo fiber photometry recordings of MS-innervated glutamatergic VTA neurons (referred to as Glu^MS–VTA^), MS-innervated dopaminergic VTA neurons (DA^MS–VTA^), and MS-innervated GABAergic VTA neurons (GABA^MS–VTA^). To achieve this, we used the anterograde trans-synaptic AAV vector (Zingg et al., 2017, 2020) together with Cre-dependent GCaMP vector (Fig. S7*A*,*D*,*G*). We found that sevoflurane GA suppressed the activity of all three VTA populations. However, they exhibited differentiated responses to different sevoflurane concentrations. Specifically, both Glu^MS–VTA^ and DA^MS–VTA^ neurons exhibited lower calcium activity during 2.4% sevoflurane compared with 1.5% sevoflurane (Fig. S7*B*,*C*,*E*,*F*). In contrast, GABA^MS–VTA^ neurons showed higher calcium activity during 2.4% sevoflurane than 1.5% sevoflurane (Fig. S7*H*,*I*). These findings indicate that MS^Vglut2^ neurons form monosynaptic and potentially functional innervations to all these three VTA populations.

### Optogenetic stimulation of MS-innervated VTA glutamatergic neurons promoted emergence from both light and deep anesthetic states

**Figure 6.**
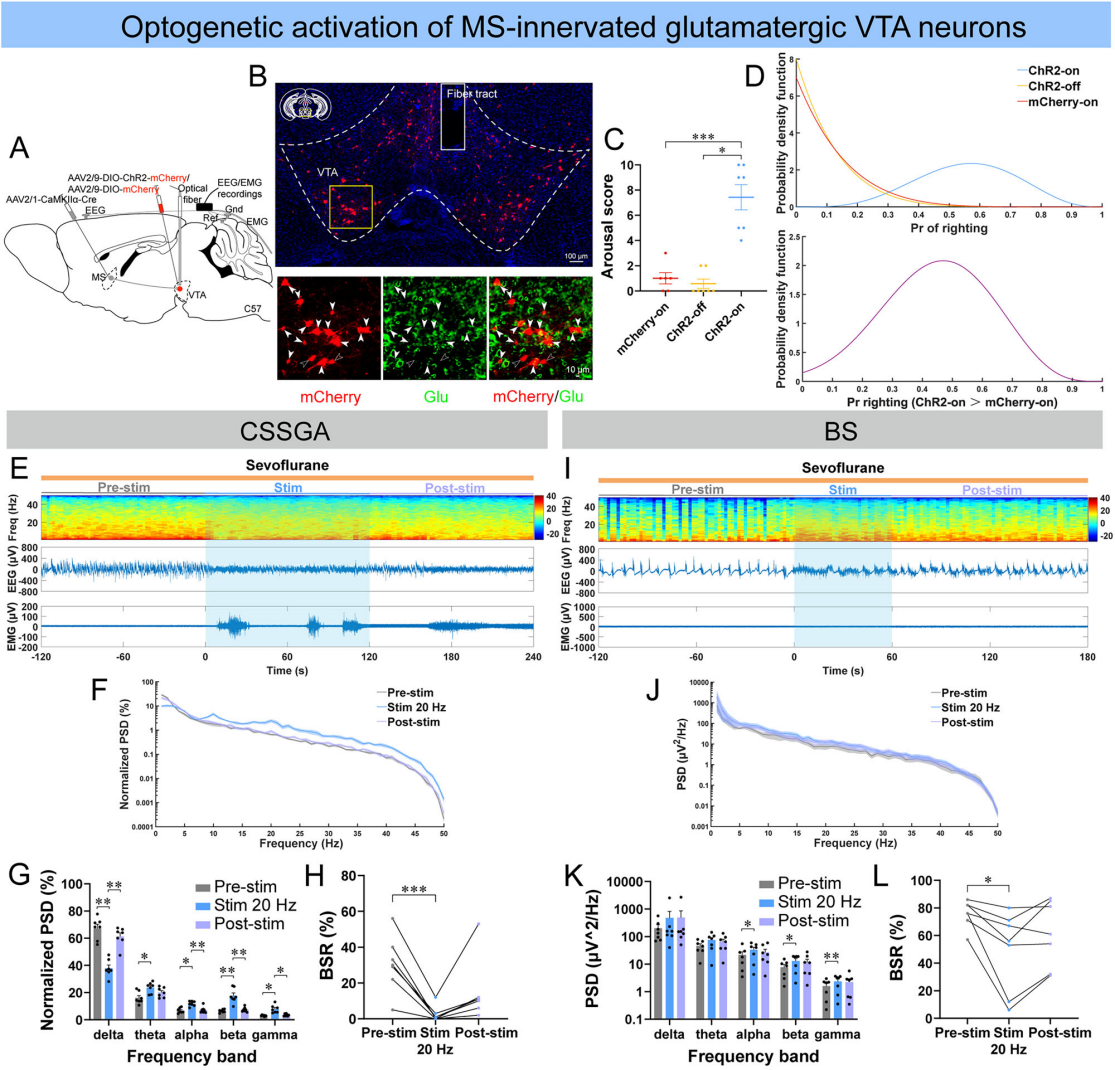
Optogenetic stimulation of MS-innervated glutamatergic VTA neurons induced behavioral arousal and cortical activation during both CSSGA and BS with sevoflurane. ***A***, The configuration of optogenetic stimulation of MS-innervated glutamatergic VTA neurons (Glu^MS–VTA^ neurons). ***B***, A representative image of the expression of ChR2-mCherry and glutamate (Glu; scale bar, top, 100 μm; bottom, 10 μm). ***C***, ***D***, Quantification of the arousal score of the three groups (***C***), posterior densities for the probabilities of righting for the three groups (***D***, top), and the difference in the probability of righting between the ChR2-on group and mCherry-on group (***D***, bottom) during optogenetic stimulation of Glu^MS–VTA^ neurons under sevoflurane CSSGA (*n* = 7 ChR2 mice; *n* = 6 mCherry mice). ***E*–*H***, A representative example of EEG/EMG of one mouse (***E***), normalized PSD of EEG (***F***, the shading indicates SEM), and quantitative analysis of normalized PSD (***G***) and BSR (***H***) before (gray), during (blue), and after (lavender) optogenetic stimulation of Glu^MS–VTA^ neurons during sevoflurane CSSGA (*n* = 7 mice). ***I*–*L***, Similar to ***E–H*** but for optogenetic stimulation of Glu^MS–VTA^ neurons during stable BS induced by sevoflurane (*n* = 7 mice). Statistical comparisons of the arousal score were conducted using Wilcoxon signed-rank test (***C***, ChR2-on vs ChR2-off), unpaired two-tailed Student's *t* test (***C***, mCherry-on vs ChR2-on), or Mann–Whitney rank-sum test (***C***, mCherry-on vs ChR2-off). Statistical comparisons of PSD or BSR were conducted using Friedman tests followed by Dunn's post hoc comparison tests (***H***), one-way (***L***) or two-way (***G***, ***K***) repeated–measure ANOVA followed by Tukey's post hoc test. *p < 0.05; **p < 0.01; ***p < 0.001. All error bars represent ±SEM.

We further conducted selective optogenetic stimulation of Glu^MS–VTA^ neurons using the anterograde trans-synaptic AAV vector and Cre-dependent ChR2 vector (Fig. 6*A*,*B*). Optogenetic stimulation of Glu^MS–VTA^ neurons by 20 Hz blue laser successfully induced behavioral arousal in ChR2 mice, including head, legs, and tail movements (7/7), righting (4/7), and walking (2/7; Fig. 6*C*; Movie 6). The Bayesian 95% CI for the difference in the probability of righting between the ChR2-on group and mCherry-on group was 0.059–0.777, and the posterior probability of the difference in righting between the two groups >0 was 0.9872, which was statistically significant (Fig. 6*D*). In addition, optogenetic stimulation of Glu^MS–VTA^ neurons also induced prominent cortical activation during both CSSGA and BS with sevoflurane, generally characterized by increased power of fast waves (including alpha, beta, and gamma) and decreased BSR (Fig. 6*E*–*L*; Movies 6, 7), which cannot be observed in control mice (Fig. S8*A*–*F*).

**Movie 6.** Optogenetic stimulation of Glu^MS–VTA^ neurons induced behavioral arousal and cortical activation during sevoflurane CSSGA in a ChR2 mouse. [View online]

**Movie 7.** Optogenetic stimulation of Glu^MS–VTA^ neurons induced cortical activation during sevoflurane BS in a ChR2 mouse. [View online]

### Optogenetic stimulation of MS-innervated VTA dopaminergic neurons promoted emergence from light anesthetic states

**Figure 7.**
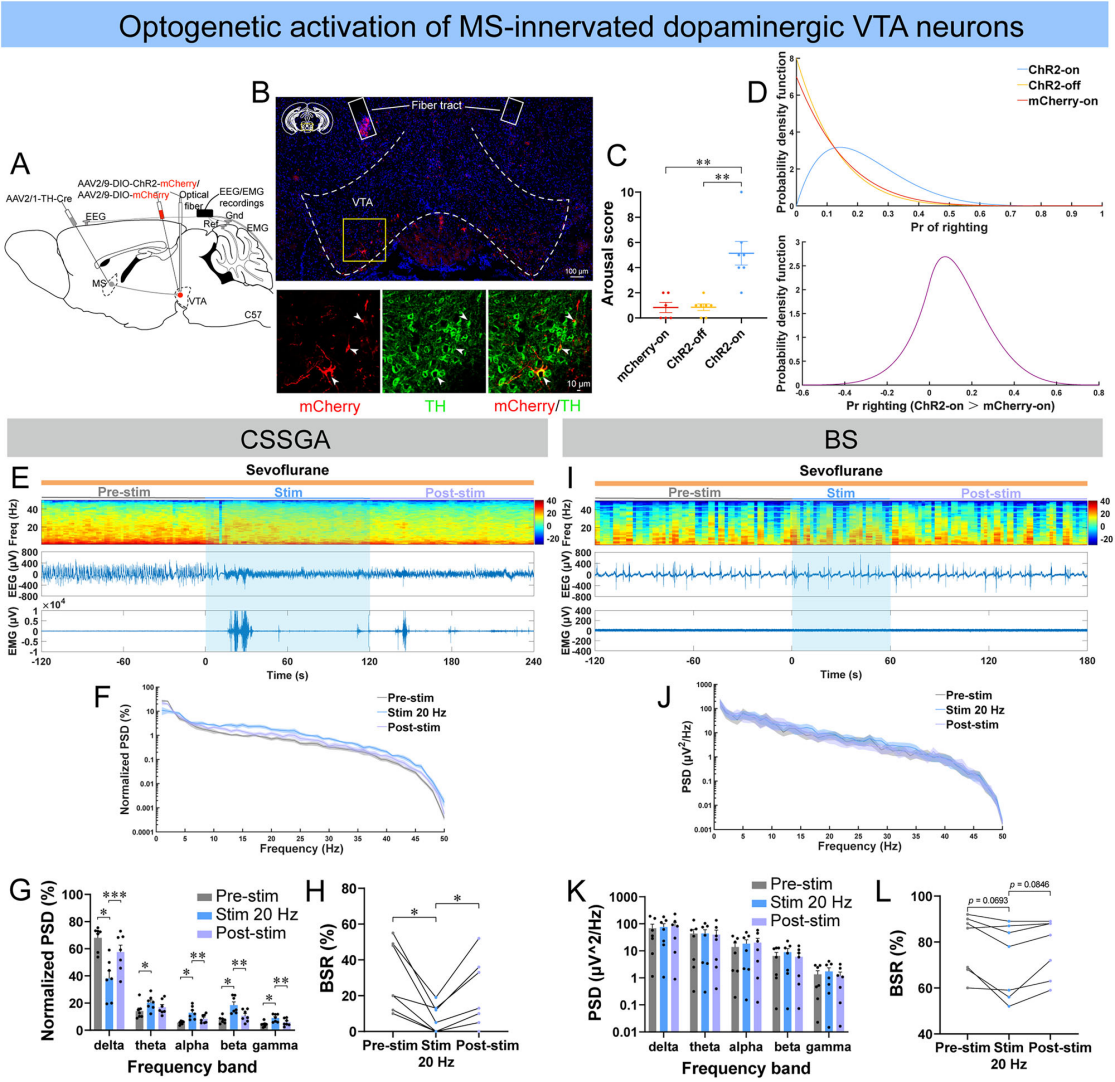
Optogenetic stimulation of MS-innervated dopaminergic VTA neurons induced cortical activation only during CSSGA with sevoflurane. ***A***, The configuration of optogenetic stimulation of MS-innervated dopaminergic VTA neurons (DA^MS–VTA^ neurons). ***B***, A representative image of the expression of ChR2-mCherry and tyrosine hydroxylase (TH; scale bar: top, 100 μm; bottom, 10 μm). ***C***, ***D***, Quantification of the arousal score of the three groups (***C***), posterior densities for the probabilities of righting for the three groups (***D***, top), and the difference in the probability of righting between the ChR2-on group and mCherry-on group (***D***, bottom) during optogenetic stimulation of DA^MS–VTA^ neurons under sevoflurane CSSGA (*n* = 7 ChR2 mice; *n* = 6 mCherry mice). ***E*–*H***, A representative example of EEG/EMG of one mouse (***E***), normalized PSD of EEG (***F***, the shading indicates SEM), and quantitative analysis of normalized PSD (***G***) and BSR (***H***) before (gray), during (blue), and after (lavender) optogenetic stimulation of DA^MS–VTA^ neurons during sevoflurane CSSGA (*n* = 7 mice). ***I*–*L***, Similar to ***E*–*H*** but for optogenetic stimulation of DA^MS–VTA^ neurons during stable BS induced by sevoflurane (*n* = 7 mice). Statistical comparisons of the arousal score were conducted using paired two-tailed Student's *t* test (***C***: ChR2-on vs ChR2-off) or Mann–Whitney rank-sum test (***C***, mCherry-on vs ChR2-off and mCherry-on vs ChR2-on). Statistical comparisons of PSD or BSR were conducted using one-way (***H***, ***L***) or two-way (***G***, ***K***) repeated–measure ANOVA followed by Tukey's post hoc test. *p < 0.05; **p < 0.01; ***p < 0.001. All error bars indicate ±SEM.

Selective optogenetic stimulation of DA^MS–VTA^ neurons during CSSGA also induced behavioral arousal in ChR2 mice, but only one out of seven ChR2 mice successfully exhibited righting and walking (Fig. 7*A*–*C*; Movie 8). The Bayesian 95% CI for the difference in the probability of righting between the ChR2-on group and mCherry-on group was −0.215 to 0.452, and the posterior probability of the difference in righting between the two groups was 0.7667 (Fig. 7*D*). During CSSGA, optogenetic stimulation of DA^MS–VTA^ neurons also induced cortical activation and increased EMG activity (Fig. 7*E*; Movie 8). Optogenetic stimulation of DA^MS–VTA^ neurons during CSSGA decreased delta power, increased the power from theta to gamma, and decreased BSR (Fig. 7*E*–*H*). However, to our surprise, optogenetic stimulation of DA^MS–VTA^ neurons during BS did not induce evident change in EEG states or EMG activity, and no significant change in PSD or BSR was found (Fig. 7*I*–*L*; Movie 9). As control, no significant difference in PSD or BSR was found in control mice during either CSSGA or BS (Fig. S8*G*–*L*).

**Movie 8.** Optogenetic stimulation of DA^MS–VTA^ neurons induced behavioral arousal and cortical activation during sevoflurane CSSGA in a ChR2 mouse. [View online]

**Movie 9.** Optogenetic stimulation of DA^MS–VTA^ neurons had little effect on the behavior or cortical state of a ChR2 mouse during sevoflurane BS. [View online]

### Optogenetic stimulation of MS-innervated VTA GABAergic neurons facilitated cortical inhibition under deep anesthetic states

**Figure 8.**
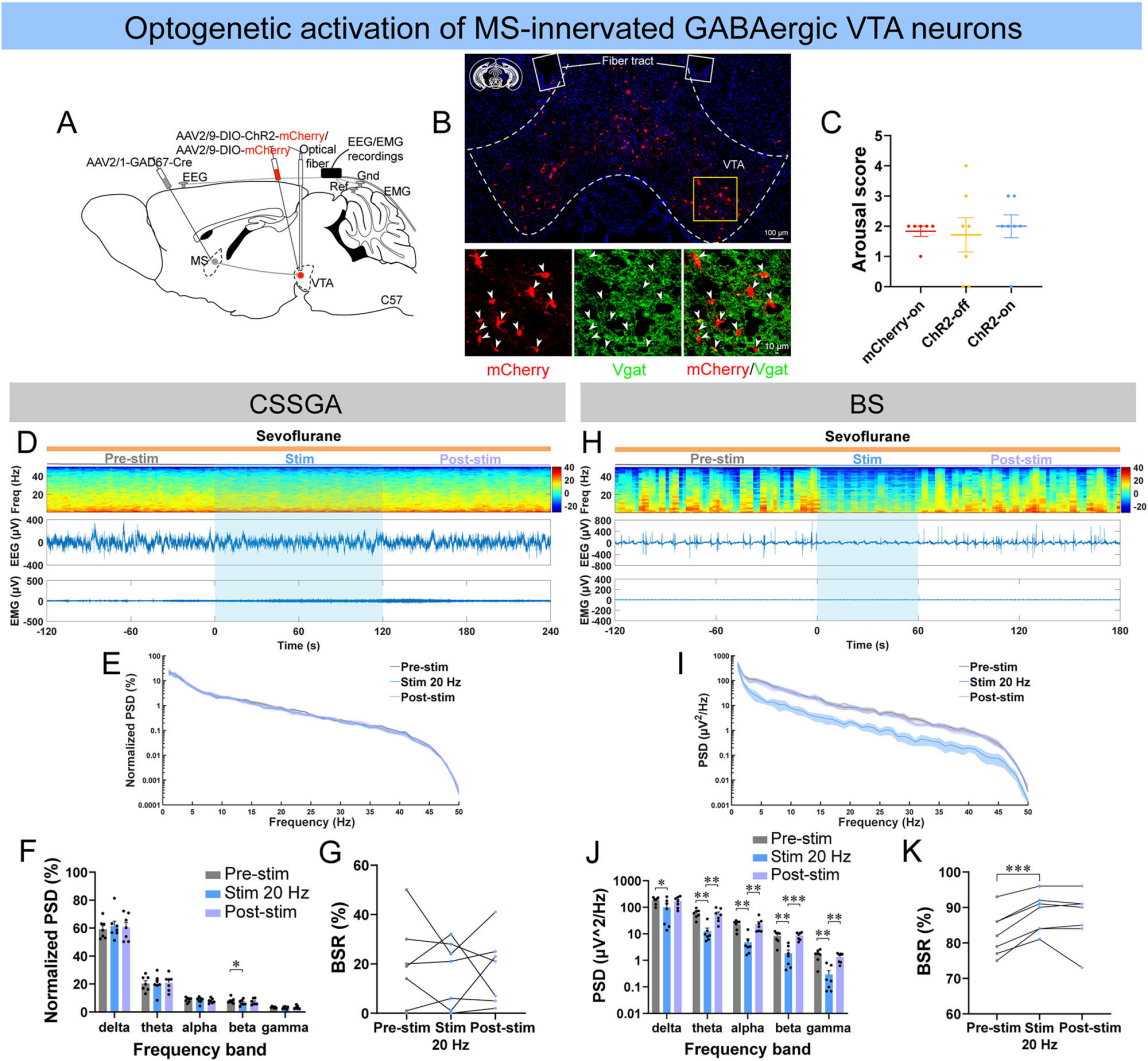
Optogenetic stimulation of MS-innervated GABAergic VTA neurons deepened cortical inhibition mainly during BS induced by sevoflurane. ***A***, The configuration of optogenetic stimulation of MS-innervated GABAergic VTA neurons (GABA^MS–VTA^ neurons). ***B***, A representative image of the expression of ChR2-mCherry and vesicular GABA transporter (Vgat; scale bar: top, 100 μm; bottom, 10 μm). ***C***, Quantification of the arousal score of the three groups during optogenetic stimulation of GABA^MS–VTA^ neurons under sevoflurane CSSGA (*n* = 7 ChR2 mice; *n* = 6 mCherry mice). ***D–G***, A representative example of EEG/EMG of one mouse (***D***), normalized PSD of EEG (***E***, the shading indicates SEM), and quantitative analysis of normalized PSD (***F***) and BSR (***G***) before (gray), during (blue), and after (lavender) optogenetic stimulation of DA^MS–VTA^ neurons during sevoflurane CSSGA (*n* = 7 mice). ***H–K***, Similar to ***D–G*** but for optogenetic stimulation of GABA^MS–VTA^ neurons during stable BS induced by sevoflurane (*n* = 7 mice). Statistical comparisons of the arousal score were conducted using Wilcoxon signed-rank test (***C***, ChR2-on vs ChR2-off) or Mann–Whitney rank-sum test (***C***, mCherry-on vs ChR2-off and mCherry-on vs ChR2-on). Statistical comparisons of PSD or BSR were conducted using one-way (***G***, ***K***) or two-way (***F***, ***J***) repeated–measure ANOVA followed by Tukey's post hoc test. *p < 0.05; **p < 0.01; ***p < 0.001. All error bars represent ±SEM.

To test whether the MS^Vglut2^–VTA^Vgat^ circuit functions in the regulation of states of consciousness during sevoflurane GA, we next conducted optogenetic stimulation of GABA^MS–VTA^ neurons (Fig. 8*A*,*B*). No significant behavioral arousal was observed during CSSGA (Fig. 8*C*; Movie 10). Besides, no significant EEG states transition or BSR change was observed, but only decreased beta power was found (Fig. 8*D*–*G*). However, it's worth noting that, during BS, optogenetic stimulation of GABA^MS–VTA^ neurons induced profound isoelectric activity, caused evident inhibition of the power from delta to gamma, and significantly increased BSR (Fig. 8*H*–*K*; Movie 11). In control mice, the same blue laser did not cause significant difference in PSD or BSR during either CSSGA or BS (Fig. S8*M*–*R*).

**Movie 10.** Optogenetic stimulation of GABA^MS–VTA^ neurons had limited effect on the cortical state of a ChR2 mouse during sevoflurane CSSGA. [View online]

**Movie 11.** Optogenetic stimulation of GABA^MS–VTA^ neurons induced profound suppression on the cortical state of a ChR2 mouse during sevoflurane BS. [View online]

Overall, optogenetic stimulation of different MS-innervated neuronal populations in the VTA induces differentiated effects on states of consciousness, which is also subject to the anesthetic state. Optogenetic stimulation of Glu^MS–VTA^ neurons causes behavioral arousal and cortical activation during both light and deep anesthetic states; optogenetic stimulation of DA^MS–VTA^ neurons induces cortical activation mainly during light anesthetic states; optogenetic stimulation of GABA^MS–VTA^ neurons enhances cortical inhibition mainly during deep anesthetic states.

## Discussion

**Figure 9.**
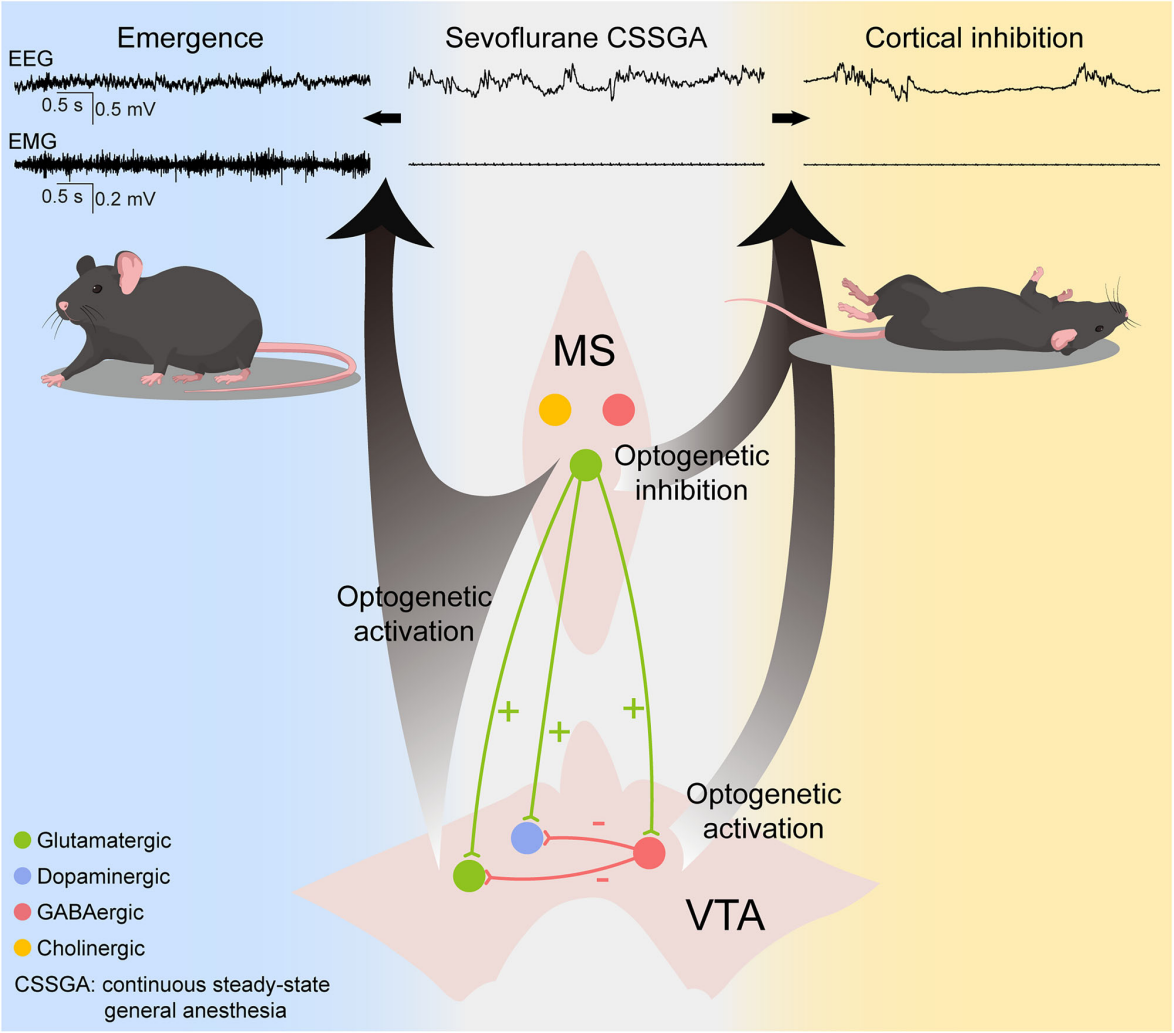
Schematic showing the heterogeneous MS–VTA circuits for regulation of states of consciousness during sevoflurane GA.

In this study, we revealed that the emergence-promoting MS^Vglut2^ neurons bidirectionally regulate states of consciousness during sevoflurane GA via projections to heterogeneous VTA neurons (Fig. 9). Specifically, optogenetic stimulation of Glu^MS–VTA^ neurons promoted emergence from both CSSGA and BS; DA^MS–VTA^ neurons facilitated cortical activation mainly during CSSGA; GABA^MS–VTA^ neurons deepened cortical inhibition mainly during BS. To our knowledge, this is the first evidence that a single neuronal ensemble can exert bidirectional regulation of states of consciousness through heterogeneous downstream circuits. Furthermore, we demonstrated that the functions of MS–VTA circuits are anesthetic state-dependent, expanding our understanding of the complex neural regulation of the consciousness level during GA.

MS^Vglut2^ neurons are well established as wake-active and wake-promoting (An et al., 2021). Previous nonspecific studies also link MS to regulation of the consciousness level during GA (Leung et al., 2013; Tai et al., 2014), but the specific functional neuronal populations remained unclear. Here, we identified MS^Vglut2^ neurons as GA-inhibited and emergence-promoting, consistent with a recent study (Xia et al., 2024). Our findings contribute to a better understanding of coordinative roles of MS^Vglut2^ neurons in regulating sleep–wakefulness and GA-induced unconsciousness.

We further demonstrated that activating MS^Vglut2^–VTA projections induced cortical activation during sevoflurane GA. Glutamatergic and dopaminergic VTA neurons promote wakefulness as well as emergence from isoflurane GA (Eban-Rothschild et al., 2016; Taylor et al., 2016; Yu et al., 2019; Zhang et al., 2024), while GABAergic VTA neurons enhance NREM sleep and isoflurane-induced unconsciousness (Chowdhury et al., 2019; Yin et al., 2019; Yu et al., 2019). Therefore, we initially hypothesized that glutamatergic and/or dopaminergic VTA neurons mediate the emergence-promoting effect of MS^Vglut2^ neurons. However, retrograde tracings of MS^Vglut2^ neurons showed their direct monosynaptic projections to all three VTA neurons.

Notably, all three MS-innervated VTA populations regulate states of consciousness during sevoflurane GA: Glu^MS–VTA^ or DA^MS–VTA^ neuronal stimulation facilitated emergence, whereas GABA^MS–VTA^ neuronal stimulation enhanced cortical inhibition. Several factors may explain these seemingly contradictions. Firstly, the emergence-promoting effect of nonspecific MS^Vglut2^–VTA axonal activation might result from combined downstream effects, where robust cortical activation from Glu^MS–VTA^ and DA^MS–VTA^ predominates over GABA^MS–VTA^ neuronal inhibitory effect. This superposition may relate to connection efficiency of these three MS–VTA circuits, warranting further investigation. Secondly, GABAergic VTA neurons promote NREM sleep partially via local projections to glutamatergic and dopaminergic neurons (Yu et al., 2019). Therefore, in our study, nonspecific MS^Vglut2^–VTA axonal activation likely influenced functional dynamics among interconnected VTA populations, leading to effects distinct from subpopulation-specific activation. Future studies should elucidate how long-range MS inputs fine-tune local VTA circuits to regulate states of consciousness during GA. Thirdly, while anterograde trans-synaptic function of AAV1 enabled specific manipulation of MS-innervated VTA neurons (Zingg et al., 2017), upstream glutamatergic MS neuronal specificity cannot be fully guaranteed. Although our retrograde tracings confirmed that VTA-targeted MS neurons are predominantly Vglut2-positive, we cannot rule out potential projections from other MS neuronal types to VTA, which warrants further exploration.

Additionally, these MS–VTA circuits exhibited anesthetic state-dependent effects, a phenomenon rarely reported in prior studies. Specifically, Glu^MS–VTA^ neuronal stimulation facilitated cortical activation during both CSSGA and BS, DA^MS–VTA^ neuronal stimulation induced cortical activation primarily during CSSGA, and GABA^MS–VTA^ neuronal stimulation enhanced cortical inhibition mainly during BS. Notably, it is reported that optogenetic activation of GABAergic LH projections to the thalamic reticular nucleus (TRN) promotes cortical activation during isoflurane-induced BS but not isoelectric activity (Herrera et al., 2016), potentially due to inhibited TRN neuronal activity under BS (Steriade et al., 1993). This suggests neuronal activity varies with anesthetic state, producing differentiated effects. Dopaminergic VTA neurons are more active during wakefulness than NREM sleep (Eban-Rothschild et al., 2016), implying higher cortical activity correlates with increased dopaminergic VTA neuronal activity. Consistent with this, we found DA^MS–VTA^ neuronal activity was higher during CSSGA than BS, explaining their ineffectiveness during BS. Conversely, our findings suggest that GABA^MS–VTA^ neurons were more excitable during BS, thus driving stronger cortical inhibition during BS. Our findings align with reports that GAD67-positive (putative GABAergic) VTA neurons are more active during NREM sleep than wakefulness (Chowdhury et al., 2019), though another study reported opposite trends for Vgat-positive VTA neurons (Yu et al., 2019). In this study, we specifically activated MS-innervated GAD67–positive VTA neurons, which may be more excitable during deeper cortical states, as reported by Chowdhury et al. The discrepancy between GAD67-positive and Vgat-positive VTA neurons requires further exploration.

Leading consciousness theories posit that brain complexity is a key element of consciousness (Sarasso et al., 2021; Q. Wang et al., 2024; Storm et al., 2024). In mice, visual detection correlates with population response heterogeneity in the primary sensory cortex (Montijn et al., 2015). Similarly, in humans, functional magnetic resonance imaging (Demertzi et al., 2019) and EEG (Frohlich et al., 2022) identify neural dynamic complexity as a common denominator of consciousness. Some theories (integrated information theory and predictive processing and neurorepresentationalism) propose that cortical connectivity and integration underpin the complexity needed for consciousness (Grasso et al., 2021; Storm et al., 2024). However, dendritic integration theory suggests long-range subcortical feedback information may impinge on the apical compartment of pyramidal neurons in layer 5 (Suzuki and Larkum, 2020; Takahashi et al., 2020). Our findings that heterogeneous MS–VTA circuits bidirectionally regulate states of consciousness in an anesthetic state-dependent manner likely reflect the underlying complexity of subcortical regulatory networks, involving distinct neural dynamics under different anesthetic states.

Our study provides some translational potentials. Firstly, unlike many previously identified circuits that only support unidirectional modulation, the MS–VTA circuits enable bidirectional control of anesthetic states. This bidirectional capability offers the potential to enhance or suppress the consciousness level via a single substrate. Secondly, the anesthetic state-dependent functional heterogeneity of this circuit provides a mechanism basis for precise, state-dependent interventions, with potential to enable individualized anesthetic depth monitoring and adjustment. Future translational efforts should focus on developing circuit-specific manipulation strategies to achieve desired clinical effects while minimizing side effects.

This study has several limitations. Firstly, we did not investigate whether sevoflurane directly acted on VTA neurons to alter the consciousness level. Given that isoflurane has been demonstrated to change consciousness level directly via VTA neurons (Taylor et al., 2016; Yin et al., 2019; Zhang et al., 2024), the direct effects of sevoflurane on VTA neuronal activity warrant further exploration. Secondly, we didn't verify the synaptic functions of these three MS–VTA circuits. Besides, the AAV1-mediated anterograde tracing strategy has inherent limitations: it (1) cannot fully guarantee the specificity of the upstream neuronal types in MS, (2) may lead to off-target labeling of ∼1–4% of downstream cells via leaky viral spread (Zingg et al., 2020), and (3) has low-level retrograde transport capability (Zingg et al., 2017, 2020). However, the concern about retrograde transport is likely minimal in our study, as previous output mapping of these three VTA populations failed to identify direct VTA–MS projections (Eban-Rothschild et al., 2016; Yu et al., 2019). Furthermore, there was a progressive increase in data dispersion of MS^Vglut2^ neuronal activity over time in the 0% group in Figure 1*D*. This phenomenon, also noted in other similar studies (Bao et al., 2021), likely reflected the inherent fluctuation of neuronal activity across natural sleep–wake cycle in unanesthetized animals. Also notice increases in baseline EEG amplitude before sevoflurane onset in Figure 1*E*, with amplitude comparable to that during post-LOC, which seems to be counterintuitive. As shown in Figure S1*B*, an example high-amplitude EEG fluctuation from this baseline correlated with elevated beta normalized power and overall power increase across all bands, which may reflect heightened cortical activities and warrants further investigation. Moreover, the righting reflex and arousal scores were used to help reflect the consciousness level, and EEG and EMG were used to assess the consciousness level in our study, which may provide a relatively comprehensive assessment. However, considering that behavior (EMG) should be distinguished from consciousness per se (Koch et al., 2016; Assadzadeh et al., 2023), further studies are still needed to establish a specific method for determining the consciousness level in mice. For method improvement, delta power in the posterior cortical regions may better correlate with consciousness (Siclari et al., 2017), and “the consciousness level index” is helpful for further research in assessing the consciousness level of mouse (Casali et al., 2013; Cavelli et al., 2023). Another concern warranting further exploration is the limited efficacy of DA^MS–VTA^ neuronal stimulation during BS, which may be due to an actual efficacy difference across anesthetic states or the shorter stimulation duration used during BS. Additionally, we only investigated these three MS–VTA circuits during the maintenance phase of sevoflurane GA, but didn't explore their roles in the induction or emergence phases.

Together, our findings unraveled bidirectional regulation of states of consciousness by heterogeneous MS^Vglut2^–VTA circuits in an anesthetic state-dependent manner. The overall characteristics of MS^Vglut2^ neurons and MS^Vglut2^–VTA projections were GA-inhibited and emergence-promoting, with MS-innervated glutamatergic VTA neurons promoting emergence during both CSSGA and BS under sevoflurane, MS-innervated dopaminergic VTA neurons mainly functioning during CSSGA to promote emergence, and MS-innervated GABAergic VTA neurons mainly functioning during BS to enhance cortical inhibition. Our findings provide new insights into the underpinnings of regulating states of consciousness during GA.

## Data Availability

The data are available from the corresponding author on reasonable request.
